# Multi-Omics Analysis of Small RNA, Transcriptome, and Degradome in *T. turgidum*—Regulatory Networks of Grain Development and Abiotic Stress Response

**DOI:** 10.3390/ijms21207772

**Published:** 2020-10-21

**Authors:** Haipei Liu, Amanda J. Able, Jason A. Able

**Affiliations:** School of Agriculture, Food & Wine, Waite Research Institute, The University of Adelaide, Urrbrae, SA 5064, Australia; amanda.able@adelaide.edu.au (A.J.A.); jason.able@adelaide.edu.au (J.A.A.)

**Keywords:** microRNAs, transcriptome, degradome, durum wheat, grain development, water deficit, heat stress, next-generation sequencing, cereal crop improvement

## Abstract

Crop reproduction is highly sensitive to water deficit and heat stress. The molecular networks of stress adaptation and grain development in tetraploid wheat (*Triticum turgidum durum*) are not well understood. Small RNAs (sRNAs) are important epigenetic regulators connecting the transcriptional and post-transcriptional regulatory networks. This study presents the first multi-omics analysis of the sRNAome, transcriptome, and degradome in *T. turgidum* developing grains, under single and combined water deficit and heat stress. We identified 690 microRNAs (miRNAs), with 84 being novel, from 118 sRNA libraries. Complete profiles of differentially expressed miRNAs (DEMs) specific to genotypes, stress types, and different reproductive time-points are provided. The first degradome sequencing report for developing durum grains discovered a significant number of new target genes regulated by miRNAs post-transcriptionally. Transcriptome sequencing profiled 53,146 *T. turgidum* genes, swith differentially expressed genes (DEGs) enriched in functional categories such as nutrient metabolism, cellular differentiation, transport, reproductive development, and hormone transduction pathways. miRNA–mRNA networks that affect grain characteristics such as starch synthesis and protein metabolism were constructed on the basis of integrated analysis of the three omics. This study provides a substantial amount of novel information on the post-transcriptional networks in *T. turgidum* grains, which will facilitate innovations for breeding programs aiming to improve crop resilience and grain quality.

## 1. Introduction

Durum wheat (*Triticum turgidum* L. *ssp. durum*) is the most economically important tetraploid wheat worldwide and is known for its extreme grain hardness, high protein content, and unique nutty flavor, making it suitable for food products like pasta [1,2]. Durum is mainly cultivated in rain-fed Mediterranean areas. Improvements in stress resilience, yield stability, and grain quality have been (and continue to be) priorities for breeding programs. The flowering and grain development processes of wheat are highly sensitive to water deficit and heat stress [3,4,5]. Upon stress, a range of physiological, molecular, and biochemical changes are induced. Examples include disrupted osmotic homeostasis, inhibited photosynthetic activities, changes in water uptake, and nutrient assimilation [3,6]. Such changes have a major impact on reproductive events such as fertilization, seed set, and grain fill, eventually affecting grain productivity, as well as quality parameters such as starch content, protein concentration, and bioactive compound concentrations [5,7]. The final impact on yield and quality may differ greatly depending on the timing, severity, and duration of stress, as well as the capability of a genotype to adapt. Moreover, water deficit and heat stress can occur independently or simultaneously under field conditions. The effects of single and combined stress could vary greatly depending on genotype [5,8]. Genotypic variation in crop performance is fundamentally underpinned by the regulatory molecular mechanisms and the possible synergistic interactions of multiple stresses [9,10]. In the era of omics technologies, knowledge-driven crop breeding now has the opportunity to generate multi-layered omics data to fully dissect such regulatory networks. The first high-quality genome of *T. turgidum* durum was released only recently [11], enabling us to harness novel information from next-generation sequencing (NGS) datasets with high accuracy and efficiency. Previous research had to either employ AABB genome sets from hexaploid wheat or perform de novo transcriptome assembly [12].

Small RNAs (sRNAs), mainly small interfering RNAs (siRNAs) and microRNAs (miRNAs), are endogenous noncoding RNAs that serve as ubiquitous epigenetic regulators [13]. sRNA-based biotechnology has emerged as one of the most powerful approaches for crop improvement [14,15]. Plant miRNAs play critical roles in modulating gene expression, by targeting sequence-specific mRNAs to induce cleavage or translation repression at the post-transcriptional level [13,15]. In crops, miRNAs have been found to regulate various biological processes, such as cellular metabolism, tissue differentiation, reproductive development, and adaptation to environmental stresses [16,17,18]. sRNA sequencing is a powerful tool for investigating conserved and novel miRNAs at the whole-genome scale [19,20,21,22,23], while degradome sequencing, the only high-throughput approach for verifying miRNA-guided mRNA cleavage sites on a genome-wide scale, has been used to validate a significant number of miRNA–target pairs in cereal crops such as hexaploid wheat, barley, rice, and maize [24,25,26,27]. Our previous report identified the mRNA degradome signatures in durum wheat flag-leaf tissue [28]. However, there has been no investigation of the *T. turgidum* miRNAome or degradome under water deficit and heat stress in developing grains. 

Here, we present the first multi-omics analysis of the sRNAome, transcriptome, and degradome in *T. turgidum* grains. We identified 606 conserved and 84 novel miRNAs at five grain developmental stages in the stress-tolerant and -sensitive genotypes. Due to the gene-silencing effect of miRNAs, the miRNA–target pairs are often antagonistically regulated (increased miRNA expression leading to reduced mRNA expression, and vice versa). Transcriptome sequencing was, therefore, used to globally capture the spatiotemporal expression patterns of mRNAs for integration with sRNA sequencing datasets and to collectively identify antagonistic expression patterns. Transcriptomic changes of 53,146 *T. turgidum* genes were described, with over 80,000 miRNA-induced mRNA cleavage sites identified via degradome sequencing. miRNA–mRNA regulatory networks related to grain development, stress adaptation, and nutrient metabolism were constructed on the basis of their annotated functions. Newly discovered DEMs (differentially expressed miRNAs) and DEGs (differentially expressed genes) involved in these networks provide new options for epi-breeding approaches aimed at improving crop resilience and grain quality. 

## 2. Results

### 2.1. Conserved and Novel miRNAs Revealed by sRNA Sequencing

A total of 118 sRNA libraries were sequenced (Table S1), from developing grain samples collected from two genotypes (DBA Aurora, stress-tolerant; L6, stress-sensitive). Four treatment groups were included, CG (control group), WS (water deficit stress), HS (heat stress), and WH (water deficit stress plus heat stress). Samples were collected at five developmental time-points (5, 15, 25, 35, and 45 DPA (days post anthesis)). Table 1 provides a summary of the abbreviations used for sample names and library names used in the paper. Over 1.28 billion raw reads were generated, from which 459.71 million clean sRNA reads were obtained. In total, 690 MIR–miRNA entries (considering both MIR gene origin and mature miRNA product) were identified (606 conserved and 84 novel), with conserved miRNAs from 52 MIR families registered in miRBase (Table S2).

**Figure 1:**
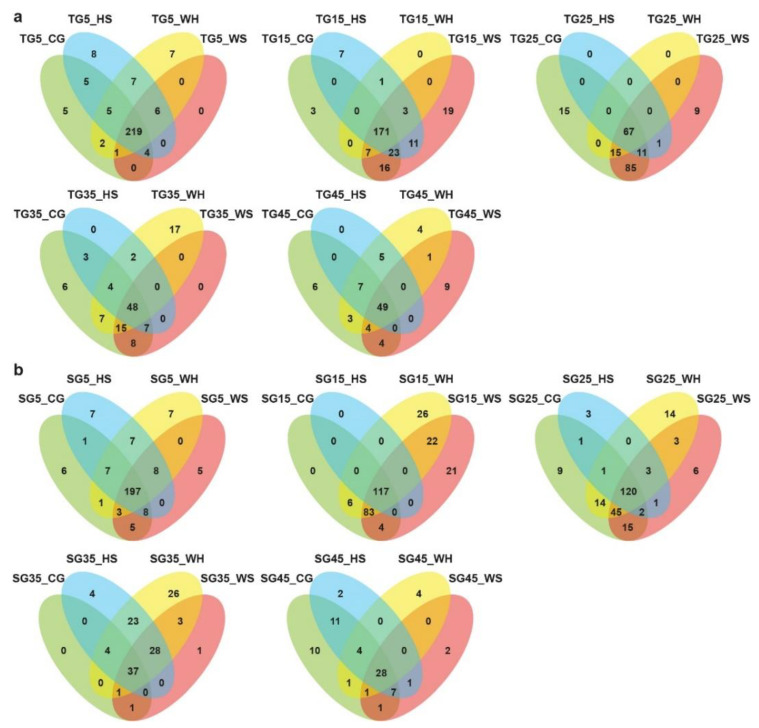
Venn diagrams showing the distribution of microRNAs across different small RNA (sRNA) sequencing libraries. (**a**) The distribution of microRNAs across four treatments at each developmental time-point in DBA Aurora libraries. (**b**) The distribution of microRNAs across four treatments at each developmental time-point in L6 libraries. TG, libraries made from developing grains of the stress-tolerant genotype DBA Aurora. SG, libraries made from developing grains of the stress-sensitive genotype L6. DPA values of 5, 15, 25, 35, and 45 indicate treatment time-points (days post anthesis). CG, control group. WS, water deficit stress group. HS, heat stress group. WH, water deficit plus heat stress group.

The distribution of miRNAs across different biological groups is shown in Figure 1 and Figure S1. Many miRNAs showed expression specificity to genotype, stress treatment, or time-point. When comparing across treatments at a time-point, there seemed to be more miRNAs exclusively expressed under WH in L6 (Figure 1b) at all time-points except for 45 DPA. For DBA Aurora (Figure 1a), the highest number of exclusively expressed miRNAs was found under HS at 5 DPA, WS at 15 and 45 DPA, CG at 25 DPA, and WH at 35 DPA. When comparing across time-points within a treatment group (Figure S1), it was evident that there were higher numbers of miRNAs exclusively expressed at early to mid-stages of grain development (5–25 DPA), irrespective of treatment and genotype. At all time-points (except for 25 DPA), DBA Aurora always had a higher number of miRNAs commonly expressed among four treatment groups when compared with L6 (Figure 1). Similar results can be found in Figure S1, where DBA Aurora always had a higher number of miRNAs commonly expressed at all DPAs.

### 2.2. DEM (Differentially Expressed miRNAs) Dependent on Stress, Genotype, and Time-Point

**Figure 2:**
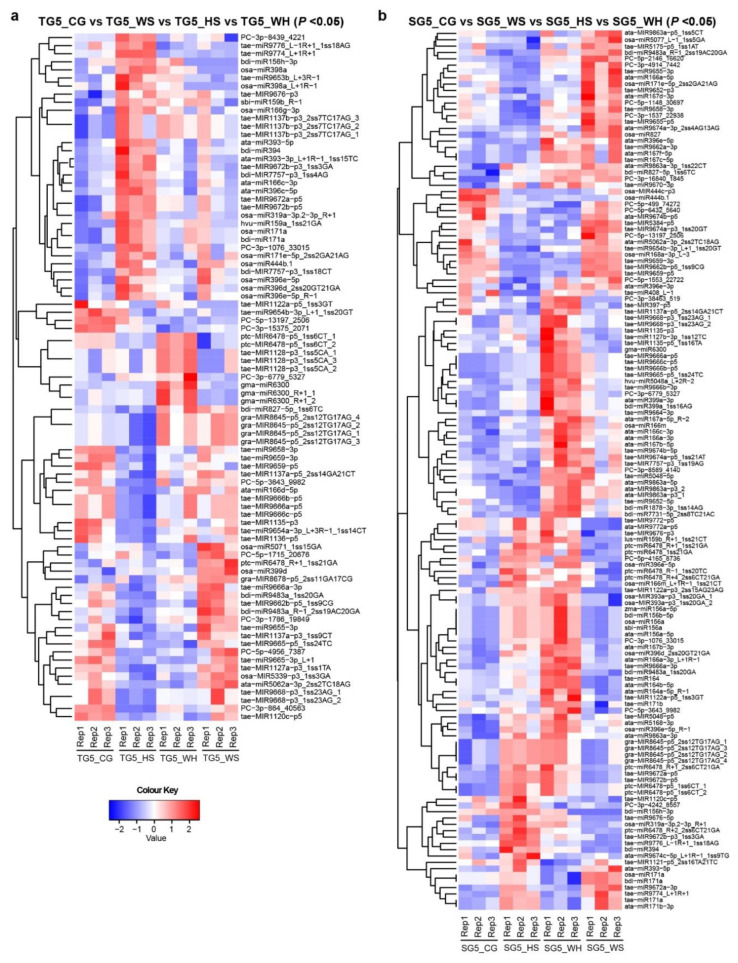
Heat maps showing differentially expressed miRNAs (DEMs) at 5 DPA across four treatment groups. (**a**) DEMs (*p* < 0.05) in DBA Aurora (TG5_CG vs. TG5_WS vs. TG5_HS vs. TG5_WH). (**b**) DEMs (*p* < 0.05) in L6 (SG5_CG vs. SG5_WS vs. SG5_HS vs. SG5_WH). The color key representing miRNA expression level is shown as the log_10_ value of the normalized read count. Figure S2 provides heat maps showing DEMs at 15–45 DPA. TG, libraries made from developing grains of the stress-tolerant genotype DBA Aurora. SG, libraries made from developing grains of the stress-sensitive genotype L6. The DPA value of 5 stands for the time-point of sampling. CG, control group. WS, water deficit stress group. HS, heat stress group. WH, water deficit plus heat stress group.

To identify DEMs in response to different stresses, miRNA expression was analyzed across treatment groups at each time-point (Table S3). Heatmaps were generated to show significant DEMs with *p* < 0.05 (Figure 2 shows DEMs at 5 DPA, Figure S2 shows DEMs at 15–45 DPA). In DBA Aurora, 85 miRNAs showed significant differential expression (*p* < 0.05) across treatments at 5 DPA, 125 miRNAs at 15 DPA, 30 at 25 DPA, 17 at 35 DPA, and 42 at 45 DPA. In L6, 139 miRNAs showed significant differential expression (*p* < 0.05) across treatments at 5DPA, 81 miRNAs at 15 DPA, 137 at 25 DPA, 35 at 35 DPA, and 16 at 45 DPA. Some miRNAs exhibited the same responsive pattern to all three stresses, while some had a distinct responsive pattern to a specific stress type (Table S3).

To identify DEMs subject to genotype, miRNA expression was compared between two genotypes under each treatment (DBA Aurora vs. L6). Upregulated DEMs represent the miRNAs that had significantly higher expression in DBA Aurora when compared with L6; downregulated DEMs represent the miRNAs that had significantly lower expression in DBA Aurora when compared with L6 (Figure S3). In most cases, the number of DEMs that were more abundant in DBA Aurora was significantly greater than the number of DEMs that were less abundant in DBA Aurora, irrespective of the *p*-value used (0.01 or 0.05). A few exceptions were found under WH at 15–35 DPA, under the CG at 25 DPA, and under HS at 25 and 35 DPA. 

To identify DEMs subject to grain development time-points, miRNA expression was compared across five DPAs under each treatment (Table S4). In DBA Aurora, 179 miRNAs showed significant differential expression (*p* < 0.05) across five time-points in the CG, 207 under WS, 176 under HS, and 143 under WH. In L6, 195 miRNAs showed significant differential expression (*p* < 0.05) across five time-points in the CG, 175 under WS, 149 under HS, and 208 under WH. The majority of these miRNAs had lower abundance in later DPAs, which is expected as the overall sRNA population tends to decline when grains reach maturity. However, some miRNAs did have significantly higher abundance during mid- to late grain development stages (Table S4). For example, in L6, the abundance of ata-miR9863a-3p peaked at 45 DPA in all treatment groups. In both genotypes, the expression of a novel miRNA, PC-3p-5230_6978, peaked at 25 DPA in all treatment groups (except for the WH group in L6).

### 2.3. Transcriptome Sequencing Identified DEGs (Differentially Expressed Genes) in Response to Different Types of Stress

We obtained over 677 million valid reads from eight transcriptome libraries constructed from 5 DPA samples (Table S1). A total of 53,146 genes and 123,292 transcript isoforms were identified, and their abundance level was normalized to the FPKM (fragments per kilobase million) standard. Figure S4 shows the abundance distribution of genes and transcripts in boxplots, with a five-number summary ordered from lowest to highest: minimum value, lower quartile, median value, upper quartile, and maximum value. The median abundance of genes in the eight libraries ranged from 2.51 to 3.14 FPKM; the mean abundance ranged from 17.28 to 19.20 FPKM. The median abundance of transcripts in the eight libraries ranged from 0.64 to 0.76 FPKM; the mean abundance ranged from 9.69 to 10.66 FPKM.

To identify DEGs with stress-responsive patterns, gene expression was compared between the CG and each stress treatment group. In DBA Aurora, 289 genes showed significant differential expression (*p* < 0.05) in response to WS, 788 genes in response to HS, and 459 genes in response to WH (Table S5). In L6, 495 genes showed significant differential expression (*p* < 0.05) in response to WS, 1673 genes in response to HS, and 1655 genes in response to WH (Table S5). Many of the genes with stress-responsive patterns are members of various transcription factor (TF) families (Table S5). Other stress-responsive genes include hormone signaling regulators and key metabolic enzymes involved in grain development (Table S5).

**Figure 3:**
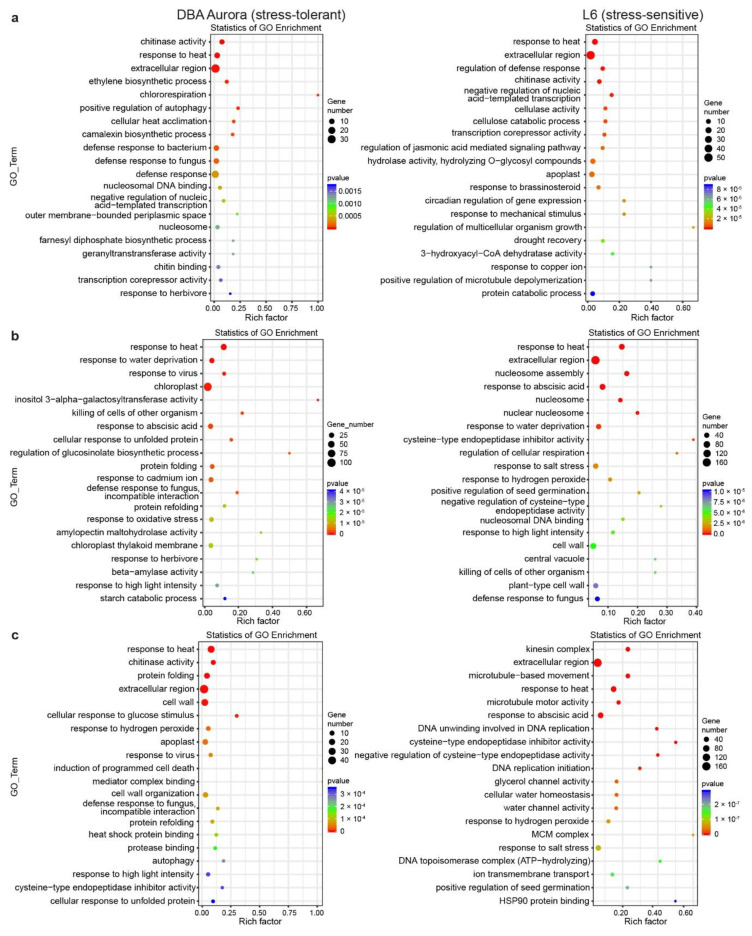
Enrichment analysis of the GO (Gene Ontology) terms for stress-responsive genes identified under each type of stress treatment. (**a**) GO analysis of genes responsive to single water deficit stress. (**b**) GO analysis of genes responsive to single heat stress. (**c**) GO analysis of genes responsive to water deficit stress plus heat stress. The rich factor represents the proportion of stress-responsive genes within all the genes under a certain GO term (higher rich factor indicates higher level of enrichment).

Gene Ontology (GO) enrichment analysis provides further information on the functions of these stress-responsive DEGs (Figure 3). Some GO terms are common to both genotypes and across stresses (for example, extracellular), while some are specific to the stress-tolerant or -sensitive genotype. Under WS (Figure 3a), GO terms such as ethylene biosynthetic process and chitin binding were only significantly enriched in DBA Aurora, while cellulose catabolic processing and response to brassinosteroids were enriched in L6. Under HS (Figure 3b), the two genotypes shared GO terms including response to abscisic acid. DBA Aurora-specific terms included cellular responses to unfolded proteins and beta-amylase activity. Highly enriched L6-specific terms included cysteine-type endopeptidase inhibitor activity and regulation of cellular respiration. Under WH (Figure 3c), the two genotypes shared terms such as response to hydrogen peroxide, while DBA Aurora-specific terms included cellular response to glucose stimulus and protease binding. L6-specific terms included cellular water homeostasis and ion transmembrane transport. 

### 2.4. Genotype-Specific Changes in DEG Expression

To identify genotype-specific expression changes, DEGs were identified between the two genotypes under each treatment condition. In total, 537 genes showed significant differential expression between DBA Aurora and L6 in the control, 263 genes under WS, 1328 genes under HS, and 1246 under WH (Table S6). The functional annotation (GO terms and the KEGG pathway classification) of these genotype-specific DEGs is provided in Table S6. The volcano plots and bar charts show the DEGs that were upregulated (more abundant in DBA Aurora vs. L6) or downregulated (less abundant in DBA Aurora vs. L6) under each treatment (Figure S5). Regardless of stress treatment, the number of DEGs that were more abundant in L6 (downregulated) was always greater than the number of DEGs that were more abundant in DBA Aurora (upregulated) under all stress treatments. The genotypic pattern of DEGs was the opposite of the pattern observed for DEMs at 5 DPA (Figure S3), which is expected considering the miRNA-induced silencing effect on the mRNA population.

GO enrichment analysis was performed for genotype-specific DEGs (Figure S6). Under control conditions, significant GO terms with a high rich factor included NAD(P)H dehydrogenase complex assembly and anion transmembrane transport. Under WS, significantly enriched terms of genotypic DEGs included cellular response to osmotic stress and histone H2B ubiquitination, while, under HS, negative regulation of cysteine-type endopeptidase activity and mitotic chromosome condensation terms were identified. Under WH, highly enriched terms included cysteine-type endopeptidase inhibitor activity, asparagine biosynthetic process, and cellular ion homeostasis. 

### 2.5. SNP (Single-Nucleotide Polymorphisms) and AS (Alternative Splicing) Analysis 

We detected 59,165 to 64,669 exonic single-nucleotide variants (SNVs), and 1116 to 1264 exonic INDELs in each transcriptome library (Table S7). Exonic SNVs and INDELs were further annotated for their effects. In each library, there was a higher occurrence of synonymous SNVs than non-synonymous SNVs. Similarly, there was a higher occurrence of stop-gain SNVs than start-loss SNVs. For INDELs in each transcriptome library, there was a higher occurrence of frameshift deletions than frameshift insertions and a higher occurrence of non-frameshift deletions than non-frameshift insertions. There were 224,272 AS events identified in the eight transcriptome libraries, as shown in Figure S7. Total numbers of alternative transcription start sites (TSS) and alternative transcription termination sites (TTS) were the highest across the 12 AS types. For all AS events, it appears that single water deficit stress reduced AS occurrence (Figure S7). On the other hand, single heat stress induced the occurrence of AS events (Figure S7). 

### 2.6. Degradome Sequencing and miRNA-Regulated mRNAs with Stress-Responsive Profiles 

Around 490.37 million raw reads were obtained from eight degradome libraries (Table S1). Of these, over 198 million reads were mapped to the reference genome (Table S1). The number of *T. turgidum durum* mRNA transcripts that could be matched in each library ranged from 144,872 to 154,448 (Table S1). Degradome signatures were further analyzed to identify mRNAs that were post-transcriptionally silenced by miRNAs as previously described [28,29]. The identified target genes of miRNAs were classified into five categories (0 to 4) as described previously [30,31], where category 0 represents mRNA targets with the highest confidence according to degradome signatures. The pairing information between miRNAs and their mRNA targets (paired and unpaired sites, complementary sequence) and the categorization of each mRNA target according to the degradomics in two genotypes can be found in Table S8. In DBA Aurora, 75,516 target transcript sites were identified with a significant differential pattern across four treatments (Table S8). In L6, 87,450 target transcript sites were identified (Table S8). To analyze miRNA-regulated mRNA targets with stress-responsive profiles, the expression levels of mRNA degradome tags were further analyzed with the GO annotation and KEGG pathway for targets with a stress-responsive degradation pattern (Table S9). In DBA Aurora, 42,464 mRNA targets showed a differential degradation pattern in response to WS; under HS, there were 41,621 targets; under WH, there were 38,634 targets. In L6, 47,557 mRNA targets showed differential degradation pattern in response to WS; under HS, there were 39,654 targets; under WH, there were 42,603 targets. To analyze miRNA-regulated mRNA targets with genotypic patterns, the expression levels of mRNA degradome tags were further analyzed between the two genotypes including with GO annotation and KEGG pathway for targets with a genotypic degradation pattern (Table S10). Under CG conditions, 44,863 targets showed a differential degradation pattern between DBA Aurora and L6; under WS, 45,485 targets showed a differential degradation pattern; under HS there were 35,484 targets; under WH, 35,558 targets showed a genotypic degradation pattern. 

### 2.7. Multi-Omics Analysis: Stress-Responsive and Genotype-Dependent miRNA–mRNA Modules 

Multi-omics analysis was performed using three types of sequencing datasets as previously described [28], to identify significant miRNA–mRNA pairs with antagonistic patterns via three steps. First, all validated miRNA–target pairs were identified on the basis of three types of sequencing data, where miRNA candidates were confirmed by sRNA sequencing, mRNA candidates were confirmed by transcriptome sequencing, and miRNA–mRNA target pairing was confirmed by degradomics. Second, from all these validated miRNA–target pairs, those with significant differential expression patterns were identified (i.e., both the miRNA and the mRNA target in the pair had to exhibit significant differential expression at *p* < 0.05, subject to the stress treatment or the genotype factor). Third, from these significant miRNA–mRNA pairs, according to the gene-silencing effect of miRNAs, the pairs with antagonistic patterns were identified, where the miRNA and the corresponding target mRNA showed an opposite regulatory pattern (i.e., significantly downregulated miRNA expression had to match significantly upregulated mRNA target expression, or significantly upregulated miRNA expression had to match significantly downregulated mRNA target expression). 

**Figure 4:**
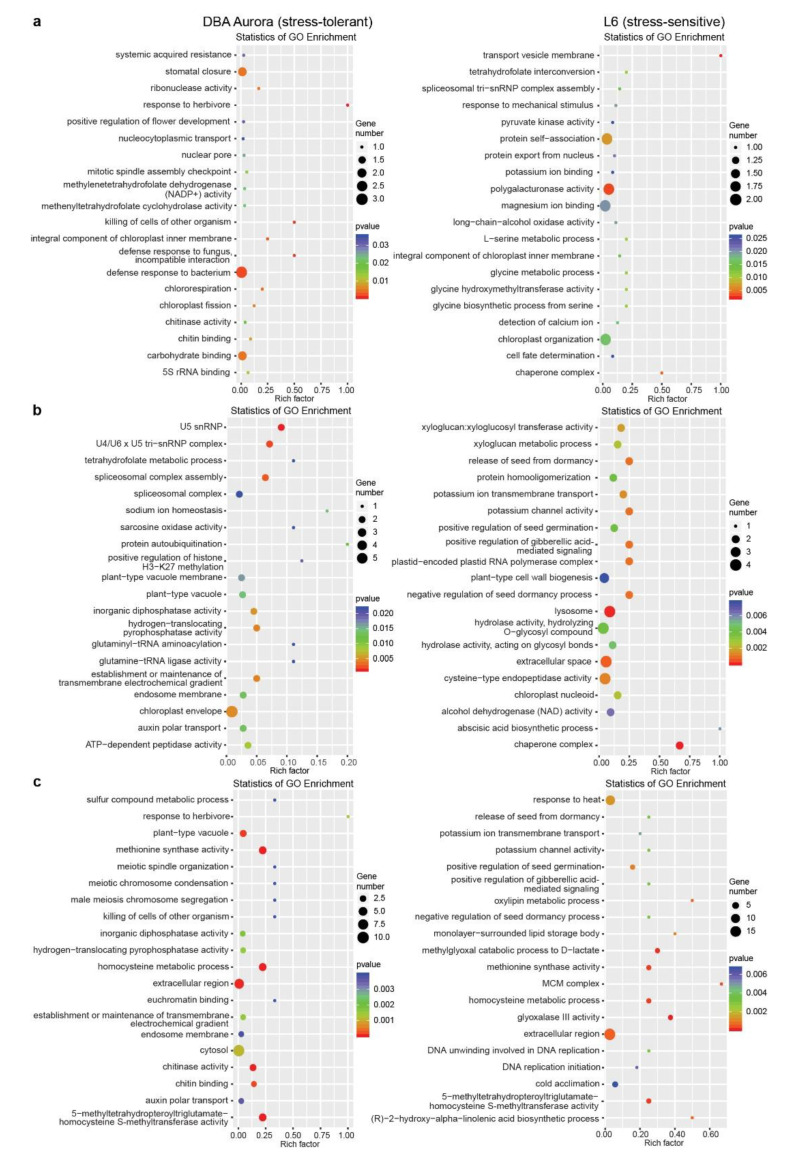
GO enrichment analysis of stress-responsive miRNA–mRNA pairs with significant (*p* < 0.05) antagonistic expression patterns subject to each stress type. (**a**) GO analysis of miRNA–mRNA pairs responsive to water deficit stress. (**b**) GO analysis of miRNA–mRNA pairs responsive to heat stress. (**c**) GO analysis of miRNA–mRNA pairs responsive to water deficit plus heat stress. The rich factor represents the proportion of stress-responsive genes within all the genes under a certain GO term (a higher rich factor indicates higher level of enrichment).

With this method, in DBA Aurora, 12 miRNA–mRNA pairs showed significant antagonistic regulatory patterns in response to WS, while there were 48 miRNA–mRNA pairs for HS and 20 miRNA–mRNA pairs for WH (Table S11, showed a differential degradation pattern). In L6, 41 miRNA–mRNA pairs showed significant antagonistic regulatory patterns in response to WS, while there were 107 miRNA–mRNA pairs for HS and 190 miRNA–mRNA pairs for WH (Table S11, showed a differential degradation pattern). Many stress-responsive genes encode proteins with critical functions in stress signaling and nutrient accumulation, such as transcription factor (TF) families NAC and MYB, peroxidases, superoxide dismutases (SODs), beta-amylases, and CBL-interacting protein kinases, with their specific functions listed in Table S11 under different stress conditions. GO enrichment analysis of these miRNA–mRNA modules revealed their biological functions under various stresses in two genotypes (Figure 4). Many of these enriched GO terms were genotype-specific. For example, under WH (Figure 4c), GO terms like auxin polar transport and chitin binding were only significantly enriched in DBA Aurora, while methionine synthase activity and oxylipin metabolic process were enriched in L6. Chitin-binding related proteins are known to generally play key roles in plant defense response, with contributions toward growth regulation, as well as abiotic stress adaptation, as shown previously where chitin-binding proteins exhibited responsive patterns to certain abiotic stresses [32,33]. Here, it was interesting that the GO term chitin binding was only enriched in the stress-tolerant genotype under WH, possibly playing a role in a genotype-specific stress response; its specific functional contribution in durum wheat, however, remains unexplored.

Table S12 shows the miRNA–mRNA pairs with genotype-dependent expression patterns under each treatment condition. Under the control condition, 69 miRNA–mRNA pairs showed antagonistic expression patterns between DBA Aurora and L6; of these, 20 pairs had higher mRNA target expression in DBA Aurora vs. L6, and 49 pairs had lower mRNA target expression in DBA Aurora. Under WS, 50 miRNA–mRNA pairs showed antagonistic expression patterns between DBA Aurora and L6; of these, 15 pairs had higher mRNA target expression in DBA Aurora vs. L6, and 35 pairs had lower mRNA target expression in DBA Aurora. Under HS, 203 miRNA–mRNA pairs showed antagonistic expression patterns between DBA Aurora and L6; of these, three pairs had higher mRNA target expression in DBA Aurora vs. L6, and 200 pairs had lower mRNA target expression in DBA Aurora. Under WH, 131 miRNA–mRNA pairs showed antagonistic expression patterns between DBA Aurora and L6; of these, 11 pairs had higher mRNA target expression in DBA Aurora vs. L6, and 120 pairs had lower mRNA target expression in DBA Aurora. It is worth noting that there were always more miRNA–mRNA pairs where mRNA expression was lower in DBA Aurora compared with L6 under all treatment conditions. The classification of KEGG pathways associated with these miRNA–mRNA modules revealed their biological roles under each treatment condition (Figure S8). 

### 2.8. qPCR Analysis of DEMs and DEGs

**Figure 5:**
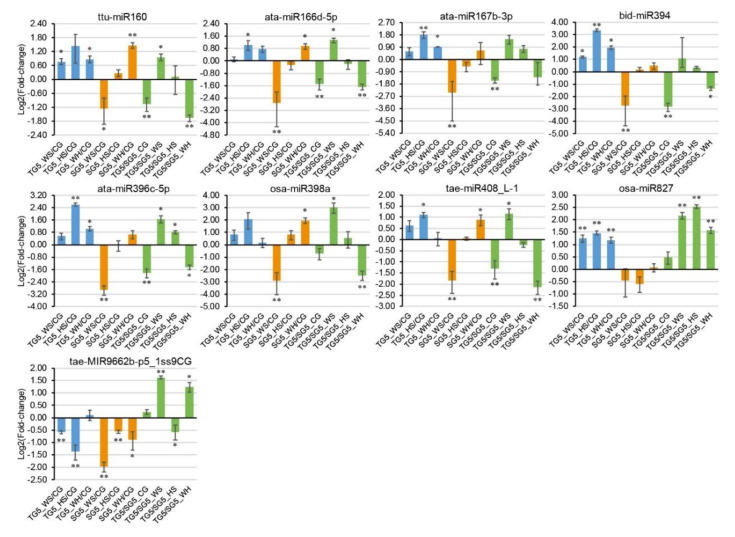
qPCR analysis of nine stress-responsive miRNAs at 5 DPA (days post anthesis). The 2^−ΔΔCt^ method was used to calculate relative miRNA expression using *GAPDH* as the housekeeping gene. Each expression profile is shown as the log_2_ value of the fold-change, and the fold-change was calculated as follows: (relative miRNA expression level under stress condition)/(relative miRNA expression level under the control condition) or (relative miRNA expression level in DBA Aurora)/(relative miRNA expression level in L6). Data are represented as the mean ± standard error (SE) (three replicates). * *p* < 0.05, ** *p* < 0.01.

**Figure 6:**
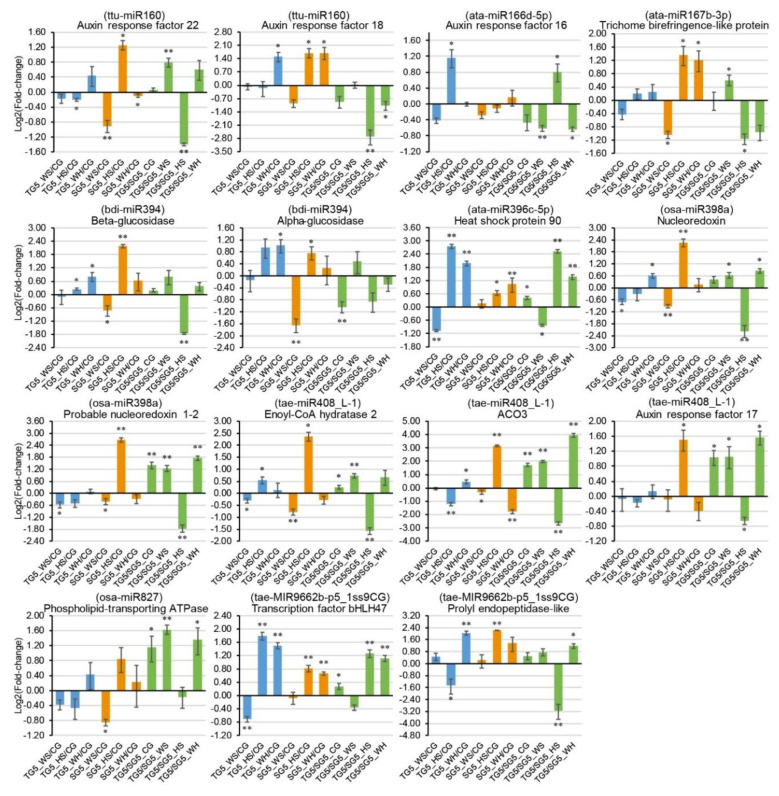
qPCR analysis of 15 stress-responsive target genes at 5 DPA (days post anthesis). The 2^−ΔΔCt^ method was used to calculate relative gene expression using *GAPDH* as the housekeeping gene. Each expression profile is shown as the log_2_ value of the fold-change, and the fold-change was calculated as follows: (relative gene expression level under stress condition)/(relative gene expression level under the control condition) or (relative gene expression level in DBA Aurora)/(relative gene expression level in L6). Data are represented as the mean ± SE (three replicates). * *p* < 0.05, ** *p* < 0.01. Abbreviations: ACO3 (1-aminocyclopropane-1-carboxylate oxidase 3).

Nine stress-responsive miRNAs and 15 stress-responsive targets were selected for qPCR analysis, according to miRNA-guided mRNA cleavage signatures validated by degradome sequencing. Genotypic patterns were observed for some of the expression profiles (Figure 5 and Figure 6). For example, osa-miR827 was significantly upregulated in DBA Aurora under all stresses but had no significant change in L6 (Figure 5). Stress-responsive miRNAs and their targets sometimes exhibited antagonistic patterns, subject to genotype and stress type. For example, in L6, tae-MIR9662b-p5_1ss9CG was downregulated in response to all stress treatments (Figure 5), and its target transcription factor bHLH47 was upregulated correspondingly under HS and WH (Figure 6). As observed in previous studies [21,34], miRNA–target expression does not always exhibit a negative correlation, as one miRNA can regulate multiple mRNAs simultaneously and one mRNA is often targeted by several miRNAs. The variation in miRNA and mRNA expression (represented as the standard deviation in figures) is not uncommon [20,35] and would have originated from the expression difference across the biological replicates used. Target t-plots (Figure S9) showed the mRNA cleavage sites within target genes silenced by miRNAs under stress.

## 3. Discussion

### 3.1. Functional Roles of T. turgidum miRNA–mRNA Modules at Different Stages of Grain Development

Rapid development of NGS techniques has led to the discovery of many conserved and novel miRNA families related to grain development in cereal crops such as bread wheat, maize, rice, and barley [36,37,38,39]. A previous study in durum also focused on miRNAs related to nitrogen starvation during grain fill, with pooled samples from root, leaf, flag leaf, and spike tissues [21]. To date, however, no research has focused on the systematic profiling of the miRNAome in *T. turgidum* developing grains at different reproductive stages. Compared with the hexaploid wheat genome (AABBDD, ~16 Gb), the *T. turgidum* genome is smaller (AABB, ~10.45 Gb), yet still considerably larger than many other cereals such as rice (~400 Mb) and maize (2.4 Gb). The size of *Triticum* genomes, therefore, poses significant challenges in the mining of NGS datasets. With our in-depth bioinformatics analysis, we successfully identified a significantly higher number of miRNA members (690 in total, of which 84 are novel), compared with previous studies in tetraploid wheat using the NGS approach. This has enabled us to fully explore the functions of conserved and novel miRNAs related to *T. turgidum* grain development.

Compared with previous research in *T. turgidum* leaf tissue [28], contrasting trends were observed between the leaf and grain miRNA populations. For grain miRNAs, DBA Aurora had a higher number of miRNAs commonly expressed in all treatment groups than that in L6 (Figure 1a,b). DBA Aurora also had a higher number of grain miRNAs commonly expressed across developmental time-points (Figure S1a,b). However, an opposite pattern was found in the leaves [28]. In the flag leaf, it was DBA Aurora that always had a lower number of commonly expressed miRNAs. These results suggest that, in the stress-tolerant genotype, the leaf miRNA population had higher expression specificity, but the grain miRNA population had lower expression specificity. A similar contrasting trend was also observed for miRNA expression level. In the grains, there were a higher number of miRNAs that had significantly higher expression in DBA Aurora vs. L6 (Figure S3). However, in the leaf, there were more miRNAs that had significantly lower expression in DBA Aurora vs. L6. Considering the different biological significance of the leaf and the developing grains, such contrasting miRNA patterns of miRNAs could be due to the different roles they play in wheat growth and development. Leaf is the major photosynthetic organ, while the most significant biological processes are related to nutrient accumulation in the grain [40,41]. A closer look into the functions of the miRNAs with contrasting expression patterns could provide more insight to their biological meaning. For example, mdm-miR397a_1ss21AT only showed significantly lower expression in the leaf tissue of DBA Aurora compared with L6, with no significant difference found in the grains. mdm-miR397a_1ss21AT targets several thylakoid lumenal proteins in the chloroplast, which serve essential roles for photosystem assembly [42]. Lower expression of mdm-miR397a_1ss21AT in DBA Aurora allowed for a higher expression of the thylakoid lumenal gene expression, which could contribute to environmental stress adaptation via protection of the photosystem [43,44]. Future research could further investigate the tissue-specific patterns of these miRNAs along with the functions of their target genes to elucidate their biological significance in wheat development and stress adaptation.

To look into the specific functions of grain miRNAs at different developmental stages, the expression dynamics of grain miRNAs were summarized into six major categories on the basis of the expression changes of miRNAs across five developmental time-points (Table S4). In two genotypes, miRNAs with a descending pattern (abundance decreased from 5 DPA to 45 DPA) had the highest percentage (ranging from 26.17% to 55.56%). In L6, miRNAs where the abundance peaked at 15 DPA had the second highest percentage. In DBA Aurora, the miRNA group with the second highest percentage had either its abundance peaking at 15 DPA or 25 DPA. miRNA groups with the lowest percentages were those with an ascending pattern (where abundance increased from 5 DPA to 45 DPA) or those with abundance peaking at 35 DPA (Table S4). Combined with the functional target information from degradome sequencing, more insight could be provided into the regulatory roles of these miRNAs with varying developmental patterns. Durum miRNAs with higher abundance at early grain development time-points seem to function mainly in cell differentiation, cell division, and hormone transduction processes that regulate embryo development and seed set (thus affecting grain number). miRNA modules with higher expression at mid to late grain development stages are involved in nutrient metabolism and transport (e.g., starch biosynthesis, nitrogen transport, and bioactive compound accumulation), which is crucial to the grain filling process (thus affecting grain size and grain quality characteristics). For example, tae-MIR167c-p3 was only detected from 5 DPA to 25 DPA in both genotypes, with the highest abundance at 5 DPA (Table S4). A newly discovered target of tae-MIR167c-p3 is a kinesin-like protein (TRITD6Av1G153400). Kinesins are microtubule-based motor proteins that are ubiquitous in all eukaryotes. Plant kinesins have direct regulatory functions in many essential cellular processes such as cell division, vesicle transport, chromosomal segregation, and cell shape determination [45,46]. The importance of the kinesin superfamily in seed formation has been demonstrated in several studies. In *Arabidopsis*, loss of a kinesin gene resulted in embryo malformation, seed abnormalities, and high abortion rates [47]. In rice, a kinesin-like protein regulates cell elongation to control grain shape and grain length, through its effects on gene expression involved in gibberellic acid synthesis [48]. The miR167–kinesin module could serve a similar role in tetraploid wheat during early reproductive stages. Further research can aim to characterize the spatiotemporal expression patterns of miR167 and kinesin genes in various young reproductive tissues, at different stages of embryonic and post-embryonic development.

### 3.2. Stress-Responsive miRNA–mRNA Modules Contribute to Stable Grain Development and Grain Quality Characteristic under Different Stresses

**Figure 7:**
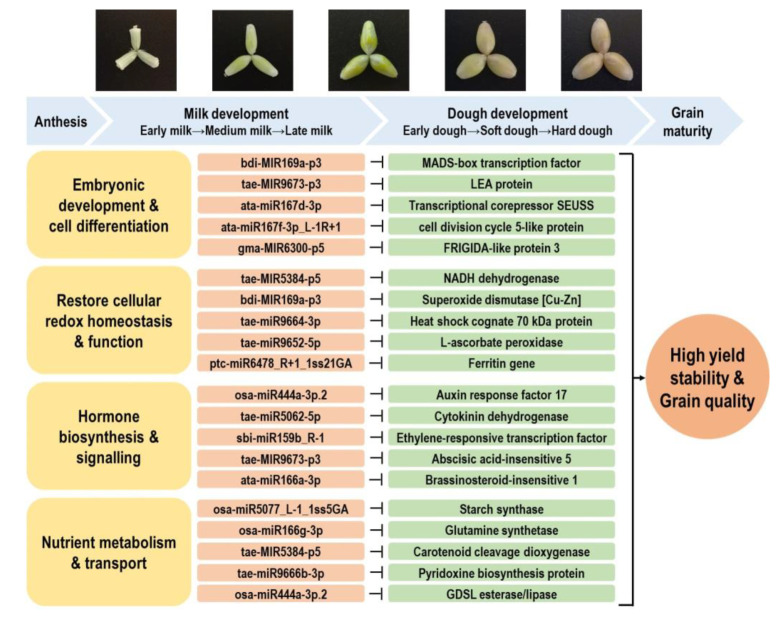
Key miRNA–RNA modules contributing to water deficit and heat stress response and grain development in durum wheat. Abbreviations: MADS domain (MCM1, AGAMOUS, DEFICIENS, SRF). LEA protein (late embryogenesis abundant protein). GDSL motif (Gly–Asp–Ser–Leu).

**Figure 8:**
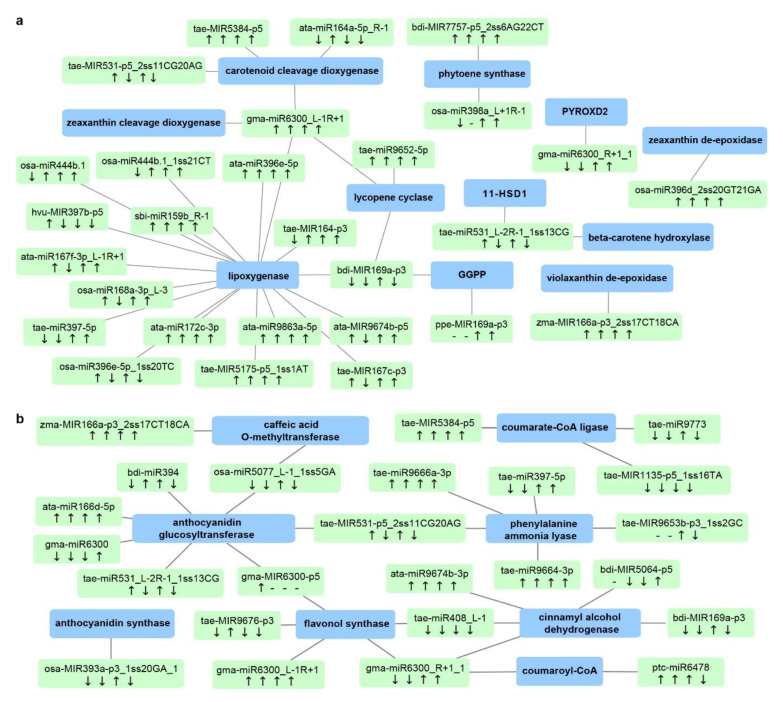
Durum miRNA–mRNA modules involved in (**a**) carotenoid biosynthesis and (**b**) the phenylpropanoid pathway. Green boxes represent miRNAs, while blue boxes represent target genes. “↓” indicates that miRNA expression was lower in the stress-tolerant genotype DBA Aurora when compared with L6; “↑” indicates that miRNA expression was higher in the stress-tolerant genotype DBA Aurora when compared with L6. The four arrows represent the miRNA/gene expression pattern under control, water deficit stress, heat stress, and water deficit plus heat stress. “-” indicates that the gene expression pattern was not available due to no expression. For genotypic differences in target gene expression pattern under the four treatment conditions, see Table S13. Abbreviations: 11-HSD1 (11-hydroxysteroid dehydrogenase type 1); GGPP (geranylgeranyl diphosphate reductase); PYROXD2 (pyridine nucleotide-disulfide oxidoreductase domain-containing protein 2). For genotypic differences in miRNA and target gene expression patterns under four treatment conditions, also see Table S13.

Grain development is one of the most critical stages which ultimately impacts the final yield obtained in cereal crops. However, this process is highly sensitive to high temperatures and declining soil water availability, which has a high occurrence in wheat-growing regions [5,49]. Our previous study demonstrated that different durum genotypes had significantly varied genotypic responses to water deficit and heat stress during reproduction [5]. Of the many genotypes studied, we demonstrated that several had a significantly higher reduction in grain number and spike fertility, while other genotypes showed high stability in fertility-related traits. Moreover, the grain quality traits (protein content, starch content, yellow pigmentation) of certain genotypes were not affected by stress [5]. The level of some beneficial bioactive compounds (e.g., phenolics) in the grains could even be induced under stress, subject to the genotype and stress type under investigation [5,7]. Such genotypic variation in yield and grain quality performance indicates that the studied durum germplasm harbors the genetic diversity that can provide resources for improving stress resilience and grain quality. Working toward this goal, we identified the stress-responsive miRNA–mRNA modules contributing to embryonic development, cellular redox homeostasis, hormone signaling, and nutrient metabolism, with examples shown in Figure 7. The construction of the miRNA–mRNA networks related to various grain nutrient components provides further information on the most promising candidates that can be considered for epi-breeding (Figure 8 and Figure S10).

Starch is the main component of the wheat endosperm, usually accounting for 55–70% of the grain weight. Starch accumulation during grain filling is the major source of grain nutrient deposition but is greatly influenced by environmental factors. In both DBA Aurora and L6, the total starch content in grain significantly decreased under water deficit and heat stress compared to the control, but with higher values observed in DBA Aurora [5]. The reduction in grain starch content is directly caused by a decrease in the expression and activity of starch synthesis-related proteins. Our research showed that many stress-responsive miRNA–mRNA modules played a part in carbohydrate metabolism, possibly contributing to a relatively stable rate of starch accumulation under water deficit and heat stress (Figure S10a). Examples include protein-coding genes such as starch synthase, glucosidase, glucan exohydrolase, amylase inhibitor, amylase, fructosyltransferase, sucrose-phosphate synthase, glucanase, and sucrose synthase. The miRNAs involved in the carbohydrate network include many conserved miRNA family members, such as tae-MIR164-p3, ata-miR166d-5p, ata-miR167b-3p, ata-miR393-5p, bdi-miR394, gma-miR6300, osa-miR396e-5p, and tae-miR408_L-1. Further investigation of the genotypic expression pattern of these miRNA–mRNA modules provided more insight into how they could contribute to the superior grain quality in the stress-tolerant genotype. For example, osa-miR5077_L-1_1ss5GA targets a *GBSSI* (granule-bound starch synthase I) gene. Under water deficit stress, this miRNA had a 2.53-fold lower expression in DBA Aurora compared with L6 (Table S12). Correspondingly, the target *GBSSI* expression was 6.74-fold higher in DBA Aurora compared with L6 (Table S12). In wheat species, grain starch is a mixture of the two major types of polysaccharides—amylose and amylopectin (ratio ~1:3). GBSSI is mainly responsible for amylose synthesis in storage tissues, but it also plays a role in the biosynthesis of extra-long unit chains in amylopectin [50]. In bread wheat, the expression of *GBSSI* gene was significantly reduced by drought stress, heat stress, and drought stress plus heat stress [51]. Interestingly, the ratio of amylose–amylopectin increased due to a higher reduction of the amylopectin content [51]. Higher expression of *GBSSI* via repressed miRNA expression in the stress-tolerant genotype could help to alleviate the negative impacts of stress on starch biosynthesis. Changes in *GBSSI* expression could also have effects on the amylose–amylopectin ratio in durum grains. There has been increasing interest in the manipulation of starch composition in durum wheat, as high-amylose flour has an increased amount of resistant starch, which provides additional health benefits for food consumption [52]. Future research could focus on investigating the expression of the osa-miR5077–*GBSSI* module and key enzyme activities in a broad germplasm panel (elite varieties and breeding lines) at multiple stages of grain development, to further explore the potential of this module in improving starch content and amylose–amylopectin composition under abiotic stress conditions.

In wheat grains, comparative proteomic analysis has shown that water deficit and heat stress usually leads to decreases in protein synthesis/assembly components and metabolism-related proteins, but the stress/defense-related proteins and seed storage proteins are often increased, especially under heat stress [53,54]. Indeed, WS, HS, and WH treatments all led to significant increases in grain protein content (GPC) in DBA Aurora. For L6, WS and WH increased the GPC, while HS had no significant impact [5]. miRNA–mRNA modules contributing to protein production are shown in Figure S10b, demonstrating the cross-regulatory relationships and the feedback loops between these stress-responsive DEMs and DEGs. Examples include storage protein assimilation-related genes such as glutamine and glutamate synthetases, as well as asparagine synthetase, avenin, glutamate receptors, and dehydrogenases (Figure S10b and Table S13). Genes encoding major determinants of end-use quality were also found, such as glutenins and gliadins. As major wheat storage proteins, the composition and ratio of glutenins and gliadins have a significant influence on the quality of pasta making [1]. Other key genes include regulators associated with post-anthesis nitrogen uptake and nitrogen use efficiency (NUE), such as nitrate transporters and nitrite reductases (Figure S10b and Table S13). The miRNAs involved in the protein network include conserved miRNAs such as ata-miR167b-3p, ata-miR172c-3p, bdi-miR394, osa-miR398a, tae-miR408_L-1, gma-MIR6300-p5, and tae-miR9652-5p. Some of these miRNA–mRNA modules exhibited stress-responsive patterns specific to DBA Aurora. For example, bdi-miR394 was downregulated under WH in DBA Aurora, and its target, a glutamate synthase gene, was correspondingly upregulated. Similarly, zma-miR156d-3p_1ss8TC was downregulated under WH in DBA Aurora, and its target, a glutamate receptor 3.5-like gene, was correspondingly upregulated. Both glutamate synthase genes (GOGAT) and glutamate receptor-like (GLR) genes are key components in plant nitrogen metabolism and protein assimilation. GOGATs act in the first step of ammonium assimilation and glutamate synthesis [55,56]. GLRs are amino-acid sensors that have roles in nitrogen and carbon sensing, with additional functions in plant defense responses and adaptation to water deficit stress [57,58,59]. In both tetraploid and hexaploid wheat, several genetic dissection studies have reported *GOGAT* and *GLR* genes localized in important quantitative trait loci (QTLs) for grain protein content, 1000-grain weight, and NUE [60,61,62]. Thus, the positive regulation of these two genes via bdi-miR394 and zma-miR156d-3p_1ss8TC under stress may be contributing to better GPC and yield performance in DBA Aurora. Notably, although stress increased the protein content for both genotypes, the GPC increase in L6 was attributed to a significant loss in the 1000-grain weight under stress. However, the 1000-grain weight was significantly increased under stress for DBA Aurora, along with its increase in GPC. The miRNA–mRNA networks constructed around protein production provides new candidates for breeding programs that aim to maximize grain protein production under water deficit and heat stress environments.

Bioactive compounds such as carotenoids and phenolics are highly valued quality traits for durum wheat. Carotenoids are a major class of natural pigments that provide the golden color (yellowness) in flour and pasta products [63]. Phenolic compounds are secondary metabolites belonging to the phenylpropanoid pathway, present in both free and bound forms in the wheat grains [7]. Both carotenoids and phenolics provide additional health benefits in whole-wheat consumption and are associated with abiotic stress adaptation in plants, mainly due to their antioxidant activities. Stress-responsive miRNAs and mRNAs associated with carotenoid biosynthesis and the phenylpropanoid pathway are shown in Figure 8. Key genes in the carotenoid network include beta-carotene hydroxylase, zeaxanthin epoxidase, phytoene synthase, lycopene cyclase, and carotenoid cleavage dioxygenase (Figure 8a and Table S13). Genes associated with the phenylpropanoid pathway include phenylalanine ammonia lyase, cinnamyl alcohol dehydrogenase, coumarate-CoA ligase, caffeic acid *O*-methyltransferase, and anthocyanidin glucosyltransferase (Figure 8b and Table S13). Many of these genes are newly reported targets regulated by miRNAs. For example, gma-miR6300_L-1R+1 seems to be a key miRNA in the carotenoid network, synergistically regulating four genes—carotenoid cleavage dioxygenase (CCD), lycopene epsilon cyclase (LCYE), zeaxanthin cleavage dioxygenase (ZCD), and lipoxygenase (LOX), all of which are newly discovered targets of the miR6300 family in *T. turgidum*. These targets function at various steps of carotenoid biosynthesis and catabolism. For example, LCYE catalyzes the biosynthesis of β-carotene, the main source of provitamin A. CCD converts larger carotenoid molecules such as β-carotene and zeaxanthin into smaller apocarotenoid products. In DBA Aurora, gma-miR6300_L-1R+1 was upregulated under WH stress and the expression of *ZCD* and *LCYE* was downregulated, while the expression of *CCD* and *LOX* remained unchanged. The results confirmed that, when one miRNA has multiple targets in the same pathway, the regulatory relationship is not always one-on-one specific. The abundance of functional genes could be fine-tuned co-operatively by a cluster of miRNAs. Further investigation into the grain quality networks could focus on such complex multiple regulatory relationships, especially the miRNA/mRNA candidates that connect throughout the pathways. Nonetheless, these identified candidates provide an entry point into improving the production of protein partitioning, as well as the coordination of unique and common biological pathways required for grain development and stress adaptation.

## 4. Materials and Methods 

### 4.1. Collection of Plant Materials and the Application of Stress

Samples were collected from two durum wheat genotypes, DBA Aurora and L6 (UAD1301020-8, University of Adelaide breeding line). According to the ranking summation index, DBA Aurora was most tolerant and L6 was most sensitive to water deficit and heat stress among previously studied Australian durum wheat genotypes [5]. The growing conditions of the plants were as described previously [5,28]. In brief, the standard control glasshouse conditions were set at 22 °C/12 °C (day/night temperature), with a photoperiod of 12 h. The light intensity averaged at 600 µmol·m^−2^·s^−1^. There were four treatment groups: CG (control), WS (water deficit stress applied pre anthesis), HS (heat stress applied post anthesis), and WH (water deficit stress plus heat stress). The details of stress application were as described previously [5,28]. Developing grain samples were collected from five developmental time-points—5, 15, 25, 35, and 45 DPA (days post anthesis). Tri reagent (Sigma-Aldrich, North Ryde, Australia) was used to extract total RNA from the developing grains. TURBO DNase was used to remove genomic DNA from total RNA samples (ThermoFisher Scientific, Scoresby, Australia). The quality, integrity, and concentration of total RNA samples were assessed with gel electrophoresis, a NanoDrop spectrophotometer, and a Bioanalyzer.

### 4.2. Small RNA Sequencing and Data Analysis

The NEBNext^®^ Multiplex Small RNA Library Prep Kit [19] was used to construct sRNA sequencing libraries (a total of 118, as listed in Table S1). The llumina NovaSeq 6000 platform was used to sequence the sRNA libraries, at the AGRF (Australian Genome Research Facility, Melbourne, Australia). All the sequencing data generated in the current study were submitted to NCBI GEO repository (GSE153932). Conserved and novel durum miRNAs were identified using the ACGT101-miR program (LC Sciences, Houston, TX, USA) as previously described [28,64]. All identified miRNAs were categorized into five groups (G1–5), with G1–4 being conserved miRNAs and G5 being novel miRNAs. DEMs were identified on the basis of the normalized read count [65], subject to genotype, treatment, and time-point. ANOVA and *t*-tests were used to identify DEMs with statistical significance (*p* < 0.05). For details, see Methods S1.

### 4.3. Sequencing and Bioinformatics Analysis of Transcriptome Libraries

For transcriptome sequencing and degradome sequencing, we used the 5 DPA time-point RNA samples with an RNA integrity number (RIN) > 9. Library construction and bioinformatics analysis were performed as previously described [28,29]. The Illumina mRNA-Seq sample preparation kit was used to construct the transcriptome libraries (eight libraries, as listed in Table S1). The Illumina NovaSeq 6000 platform was used for transcriptome sequencing at LC-Bio (Hangzhou, China). Bioinformatics analysis of transcriptome sequencing was performed as previously described [28,29]. For details, see Methods S1.

### 4.4. Sequencing and Bioinformatics Analysis of Degradome Libraries

Degradome library construction and bioinformatics analysis were performed as previously described [28,29]. The Illumina HiSeq2500 platform was used for degradome sequencing at LC-Bio (Hangzhou, China). Bioinformatics analysis of degradome sequencing was performed as previously described [28,29], using the CleaveLand software and the ACGT101-DEG package. The identified target genes of miRNAs were classified into five categories (0–4) as described previously [30,31]. For details, see Methods S1.

### 4.5. Multi-Omics Anlysis and Enrichment Analysis of GO Terms and KEGG Pathways

The enrichment analysis of GO terms and KEGG pathways was performed as described previously [28,66]. Multi-omics analysis was performed with the combination of three types of sequencing datasets as described previously [28]. Briefly, validated miRNA–target pairs were first identified, where miRNA candidates could be confirmed by sRNA sequencing, mRNA candidates could be confirmed by transcriptome sequencing, and mRNA targets could be confirmed by mRNA degradation signatures in degradome sequencing. Second, validated miRNA–mRNA pairs with significant differential expression patterns (*p* < 0.05) were identified. Third, according to the gene-silencing effect of miRNAs, significant miRNA–mRNA pairs with antagonistic expression patterns were identified.

### 4.6. qPCR Quantification of miRNAs and Target Genes with Stress-Responsive Patterns

qPCR was used to analyze the expression profile of nine miRNAs and 15 target genes with stress-responsive expression patterns. The MystiCq microRNA complementary DNA (cDNA) Synthesis Mix Kit (Sigma-Aldrich, North Ryde, Australia) was used for cDNA synthesis as described previously [28,34,67]. The PowerUp SYBR Green Master Mix (ThermoFisher Scientific, Scoresby, Australia) was used for qPCR, and the reactions were run on the ViiA7 Real-Time PCR machine with three biological replicates. The *GAPDH* gene was used as the housekeeping gene for calculating 2^−ΔΔCt^ [23,28,34]. Gene expression patterns (mean ± standard error (SE)) were shown as log_2_ fold-change of the gene abundance under stress treatment/gene abundance under the control condition. The statistical significance was determined at *p* < 0.05 (*) and *p* < 0.01 (**). Table S14 shows the primer sequences of the studied miRNAs and genes.

## 5. Conclusions

As a tetraploid species, durum wheat offers a valuable and alternative system to elucidate the stress response strategies in cereal crops, particularly when compared to bread wheat. Improving grain yield and quality under challenging environments has always been a key target for durum breeding programs. Sequence-specific recognition of miRNA–mRNAs represents a highly efficient mechanism for gene regulation in crops. In the current study, we provided the first multi-omics NGS analysis of durum miRNAs in developing grains, and the mRNAs that were precisely regulated at the post-transcriptional and transcriptional level. The expression profiles described here not only include genotypes with contrasting stress tolerance levels, but also include multiple grain developmental time-points, as well as treatment groups designed to specifically target the effects of single and combined stresses that are typically encountered during the grain filling process. The miRNA-mediated regulatory networks in *T. turgidum* grains are, no doubt, highly complex but intricately coordinated. These new findings lay the foundations for an understanding of epigenetic control to improve crop resilience through molecular breeding strategies.

## Figures and Tables

**Table 1:** Summary of the abbreviations used for sample names and library names.

Type of Terms	Abbreviations	Description
Four treatment groups	CG	Control group
	WS	Pre-anthesis water deficit group
	HS	Post-anthesis heat stress group
	WH	Pre-anthesis water deficit plus post anthesis heat stress group
Two sample types	TG	Developing grains from the stress-tolerant genotype DBA Aurora
	SG	Developing grains from the stress-sensitive genotype L6
Five time-points	5, 15, 25, 35, 45 DPA	Sampling time-points; DPA represents days post anthesis.

## Supplementary Materials

Supplementary materials can be found at https://www.mdpi.com/1422-0067/21/20/7772/s1. Supplementary Procedure. Methods S1. Details of library construction and bioinformatic analysis for the three sequencing platforms.
