# Supplementary material for: Multi-Omics Analysis of Small RNA, Transcriptome, and Degradome in T. turgidum—Regulatory Networks of Grain Development and Abiotic Stress Response
Source: Int J Mol Sci. 2020 Oct 21;21(20):7772. doi: 10.3390/ijms21207772 (PMC7589925; doi:10.3390/ijms21207772)
Supplement: Supplementary file 1 [file ijms-21-07772-s001.zip › Supplementary files Proof Part 2 out of 3/Methods S1.docx]

Methods S1. Details of library construction and bioinformatic analysis for the three sequencing platforms.

Small RNA sequencing pipeline

The NEBNext® Multiplex Small RNA Library Prep Kit was used to construct sRNA sequencing libraries following the manufacturers’ instructions [1,2]. The bioinformatics analysis of sRNA-seq was carried out using the ACGT101-miR program as described previously [1,3]. First, low quality reads and adapter sequences were removed from the raw reads. Clean reads with a nucleotide (nt) length between 18 nt and 25 nt were obtained through length filtering. Using the Rfam, Repbased and durum wheat mRNA transcripts as references, non-sRNA reads were removed, including non-coding RNAs (rRNA, tRNA, snRNA and snoRNA), repeats and mRNA sequences. The identification of conserved miRNAs and their 5p- or 3p-derived variants was performed by BLAST searching the clean sRNA-seq reads against the current registries in the plant miRBase version 22.1. Both the MIR gene sequence and the mature miRNA sequence registered in the miRBase were used as references. Single mismatch and length variation were allowed in the sequence alignment. sRNA reads that were mapped to the mature miRNA arm in the miRNA hairpin were considered as conserved mature miRNAs. sRNA reads that were mapped to the opposite arm of the mature miRNA were classified as 5p- or 3p-derived variants. The origin of MIR gene (genomic location) was determined by mapping the aligned miRNAs to the durum wheat genome assembly (Svevo.v1). After identifying the conserved miRNAs, the remaining sRNA reads were used to identify novel durum wheat miRNAs. sRNA sequences were first aligned to the durum wheat genome assembly. Secondary miRNA hairpin structures that contain the aligned sequences were identified with the RNAfold software as previously described [1,3].

The identified miRNAs were categorised into five groups (G1-5) [1,3], using the following criteria: Group 1 contain miRBase-aligned pre-miRNAs that can be mapped to the durum wheat genome and expressed sequence tags (ESTs); Group 2 contain miRBase-aligned miRNAs that can be mapped to the genome and ESTs, with their aligned genome sequences forming secondary hairpins; Group 3 contain miRBase-aligned miRNAs that can be mapped to the genome and ESTs, but their aligned genome sequences do not form secondary hairpins; Group 4 contain miRBase-aligned miRNAs that cannot be further mapped to the genome; Group 5 contain novel miRNAs that can be mapped to the genome, with their aligned genome sequences forming secondary hairpins. Reads count of miRNAs were normalised in each library to enable expression anlaysis as previously described [4].

Transcriptome sequencing pipeline

Approximately 10 µg of total RNA per sample was used for transcriptome library construction. The Illumina mRNA-Seq sample preparation kit was used to construct the transcriptome libraries as previously described [1,5]. Briefly, poly(T)-coated magnetic beads were used to enrich poly(A) mRNAs from the total RNA sample. Isolated mRNAs were fragmented and reverse-transcribed into cDNA using the Illumina mRNA-Seq kit following the manufactures’ instructions. Paired-end sequencing was carried out on the Illumina NovaSeq 6000 platform at LC-Bio (Hangzhou, China). For bioinformatics analysis, low quality reads (the ones containing adapter sequence and the ones with a sequencing quality score under 20) were removed before sequence assembly. Clean reads were then aligned to the durum wheat reference genome using HISAT version 2.0. Aligned reads from each transcriptome library were assembled. The relative transcript abundance were obtained using StringTie version 1.3.0, expressed in FPKM (Fragments Per Kilobase Million).

Degradome sequencing pipeline

Approximately 20 µg of total RNA per sample was used for degradome library construction as previously described [1,5]. mRNA was first captured from the total RNA sample with magnetic beads. The isolated mRNA fragments were annealed with biotinylated random primers. Afterwards, 5' adaptors were aligned to the mRNA fragments. First-strand cDNA was reverse-transcribed from the ligated mRNAs and was then amplified with PCR. The degradome libraries were sequenced on an Illumina Hiseq2500 at LC-Bio (Hangzhou, China). Degradome-seq reads were analysed using the ACGT101-DEG program version 4.1 (LC Sciences, USA). Low quality reads, reads with adapter and primer sequences, and non-coding RNA reads were removed. Clean reads were used to identify the degraded fragments of mRNA targets of miRNAs using the CleaveLand package version 4.0 and the ACGT101-DEG program [1,5]. Based on the ratio of the mRNA target tags relative to the overall degradome reads, the identified mRNA targets were grouped into five categories (category 0-4) as previously described [6,7]. Category 0 has the highest confidence level.

**Supplemental References**

1. Liu, H.; Able, A.J.; Able, J.A. Integrated analysis of small RNA, transcriptome and degradome sequencing reveals the water-deficit and heat stress response network in durum wheat *Int. J. Mol. Sci.* **2020**, *21*.

2. Liu, H.; Searle, I.R.; Watson-Haigh, N.S.; Baumann, U.; Mather, D.E.; Able, A.J.; Able, J.A. Genome-wide identification of microRNAs in leaves and the developing head of four durum genotypes during water deficit stress. *PLoS One* **2015**, *10*, e0142799.

3. Liu, H.; Able, A.J.; Able, J.A. Transgenerational effects of water-deficit and heat stress on germination and seedling vigour - new insights from durum wheat microRNAs. *Plants* **2020**, *9*, 189.

4. Li, X.; Shahid, M.Q.; Wu, J.; Wang, L.; Liu, X.; Lu, Y. Comparative small RNA analysis of pollen development in autotetraploid and diploid rice. *Int. J. Mol. Sci.* **2016**, *17*, 499.

5. Zhong, M.; Huang, F.; Luo, R.; Lv, Y.; Ali, U.; Sheng, Z.; Tang, S.; Wei, X.; Hu, P. The effect of cadmium on the microRNAome, degradome and transcriptome of rice seedlings. *Plant Growth Regul.* **2020**, *90*, 15-27.

6. Sun, W.; Xu, X.H.; Wu, X.; Wang, Y.; Lu, X.; Sun, H.; Xie, X. Genome-wide identification of microRNAs and their targets in wild type and phyB mutant provides a key link between microRNAs and the phyB-mediated light signaling pathway in rice. *Front. Plant Sci.* **2015**, *6*, 372.

7. Ji, Y.; Chen, P.; Chen, J.; Pennerman, K.K.; Liang, X.; Yan, H.; Zhou, S.; Feng, G.; Wang, C.; Yin, G. Combinations of small RNA, RNA, and degradome sequencing uncovers the expression pattern of microRNA-mRNA pairs adapting to drought stress in leaf and root of *Dactylis glomerata* L. *Int. J. Mol. Sci.* **2018**, *19*, 3114.
