# Supplementary figures and images for: Multi-Omics Analysis of Small RNA, Transcriptome, and Degradome in T. turgidum—Regulatory Networks of Grain Development and Abiotic Stress Response
Source: Int J Mol Sci. 2020 Oct 21;21(20):7772. doi: 10.3390/ijms21207772 (PMC7589925; doi:10.3390/ijms21207772)

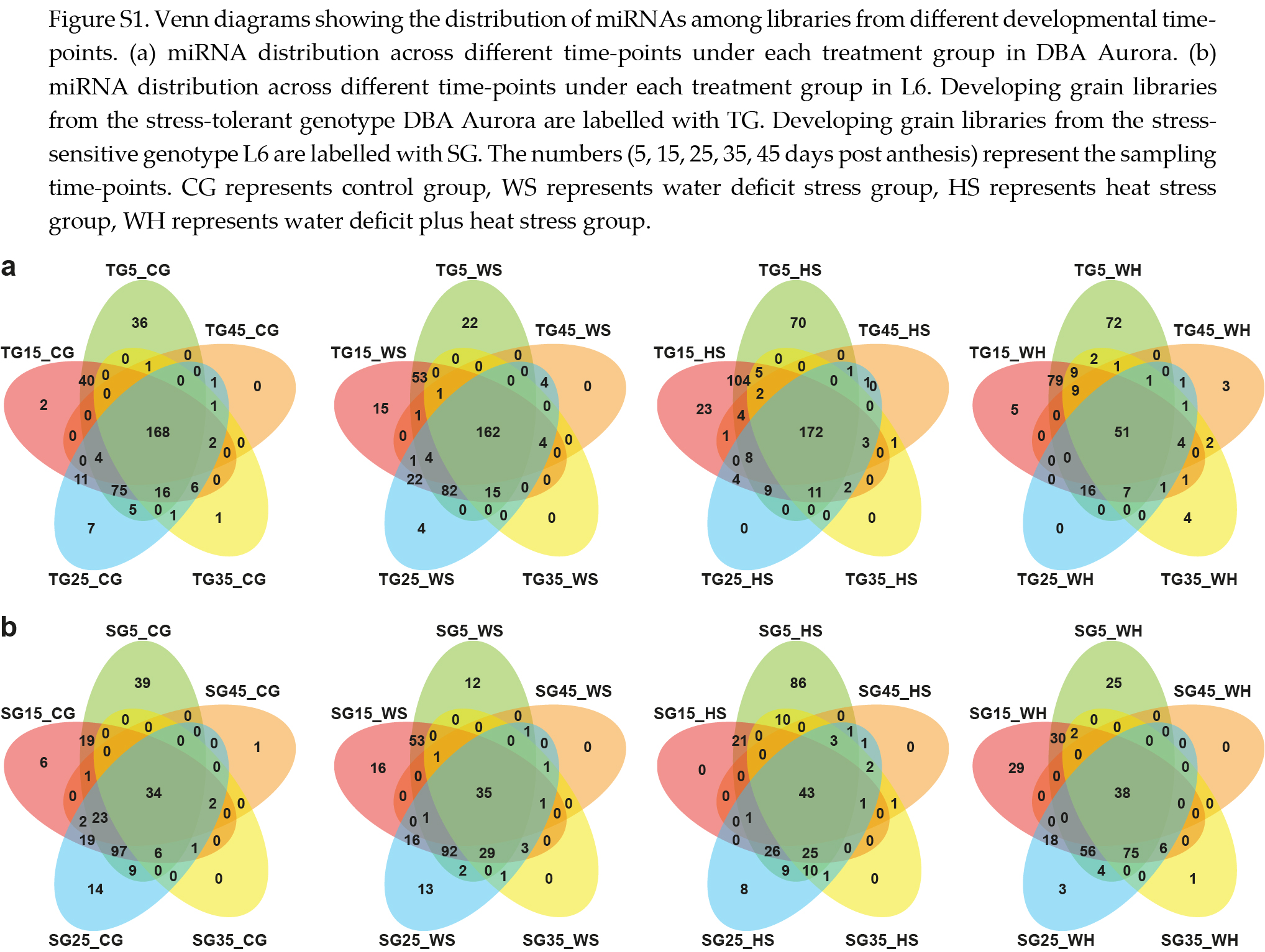

Supplement: Supplementary file 1 [file ijms-21-07772-s001.zip › Supplementary files Proof Part 1 out of 3/Figure S1.jpg]

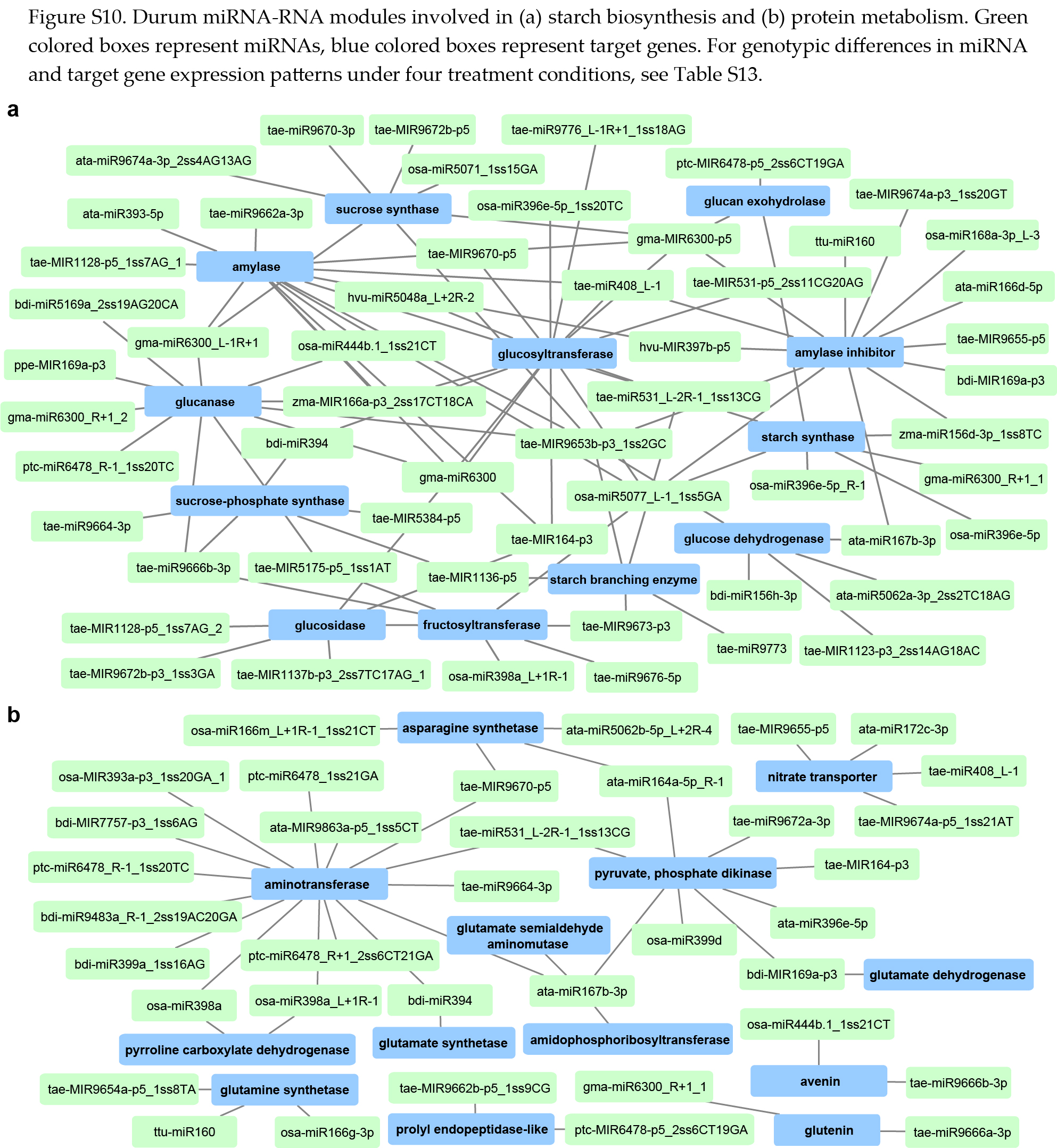

Supplement: Supplementary file 1 [file ijms-21-07772-s001.zip › Supplementary files Proof Part 1 out of 3/Figure S10.jpg]

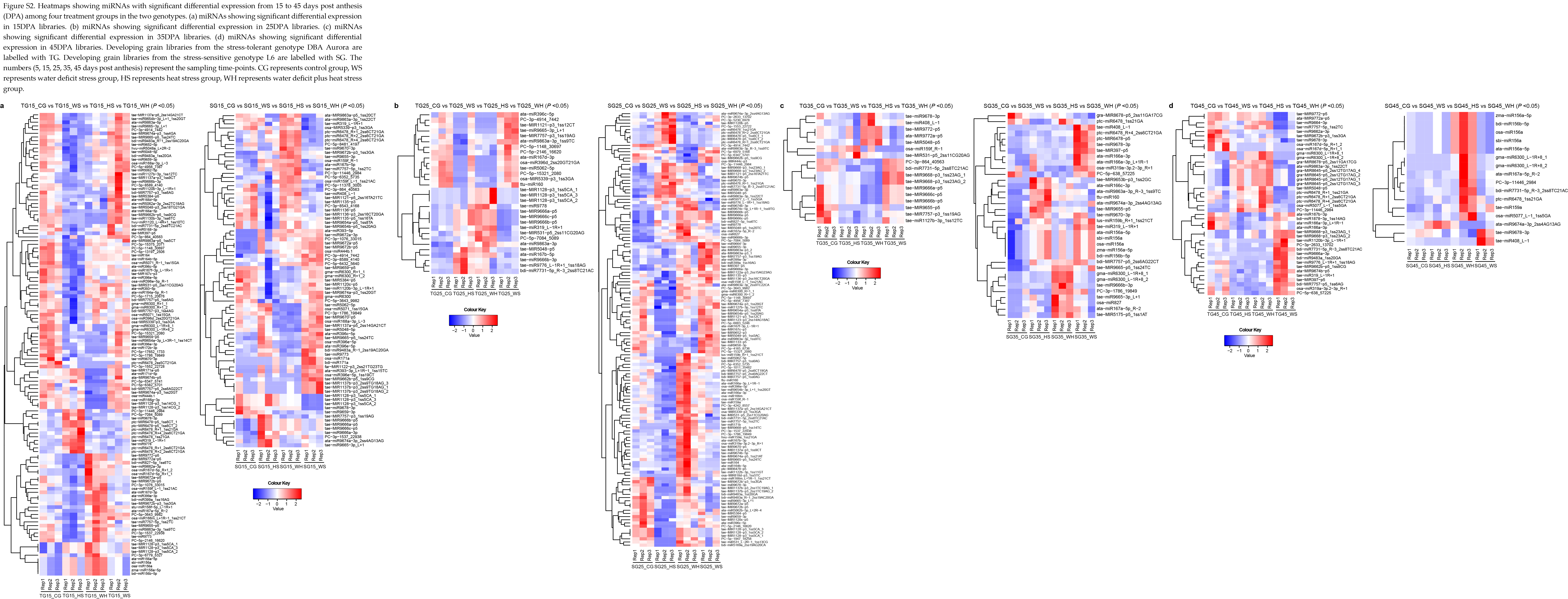

Supplement: Supplementary file 1 [file ijms-21-07772-s001.zip › Supplementary files Proof Part 1 out of 3/Figure S2.jpg]

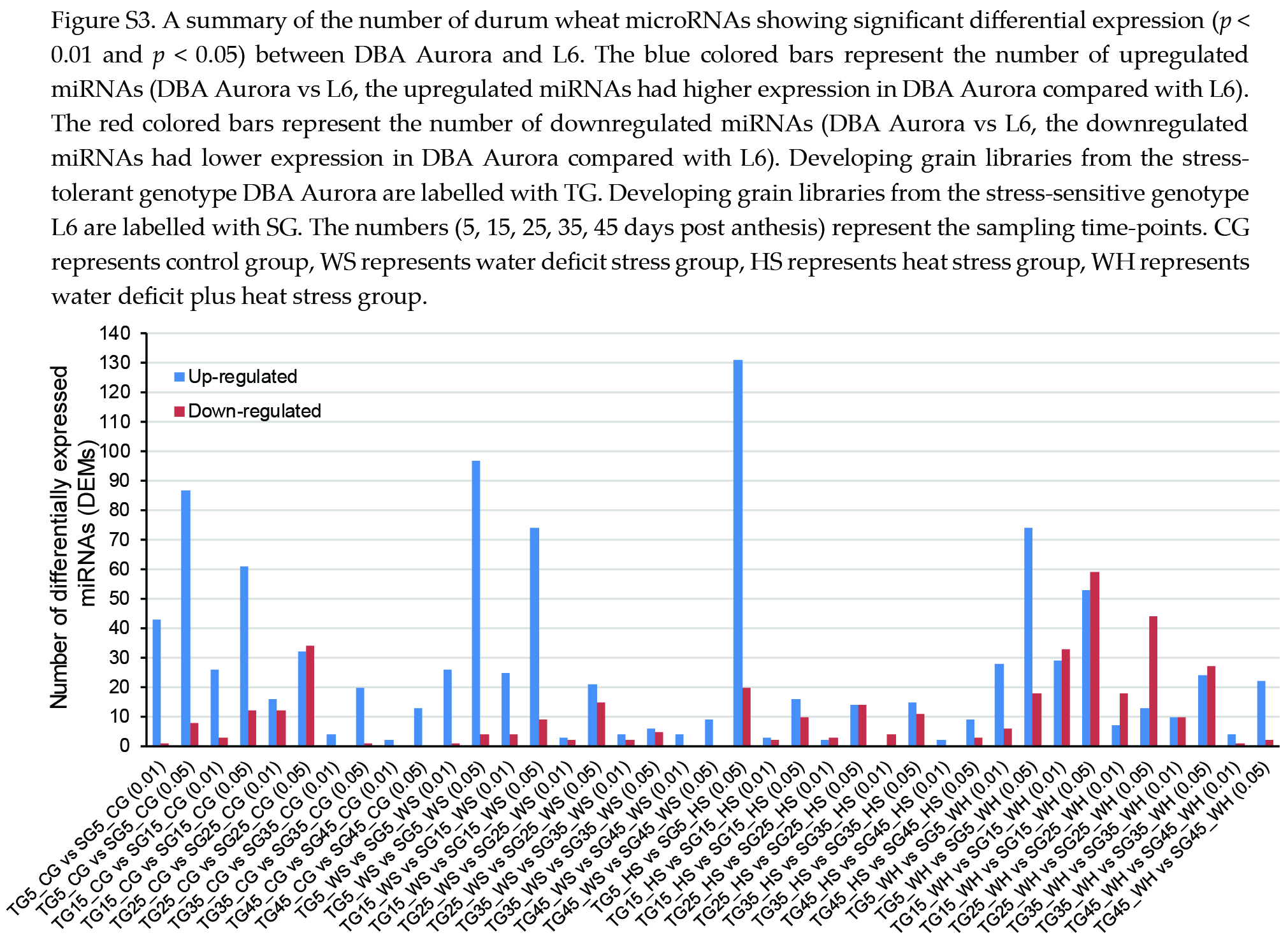

Supplement: Supplementary file 1 [file ijms-21-07772-s001.zip › Supplementary files Proof Part 1 out of 3/Figure S3.jpg]

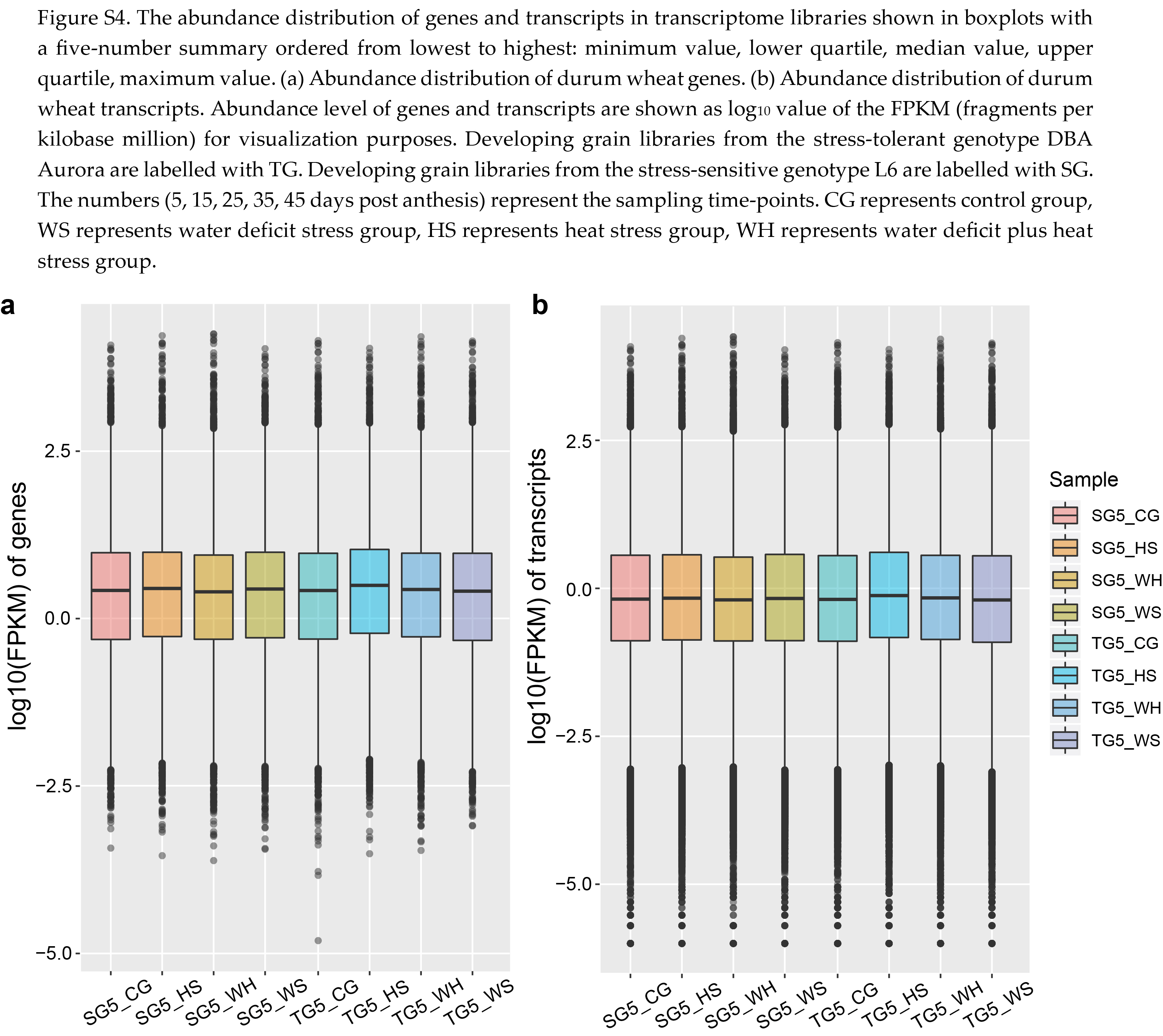

Supplement: Supplementary file 1 [file ijms-21-07772-s001.zip › Supplementary files Proof Part 1 out of 3/Figure S4.jpg]

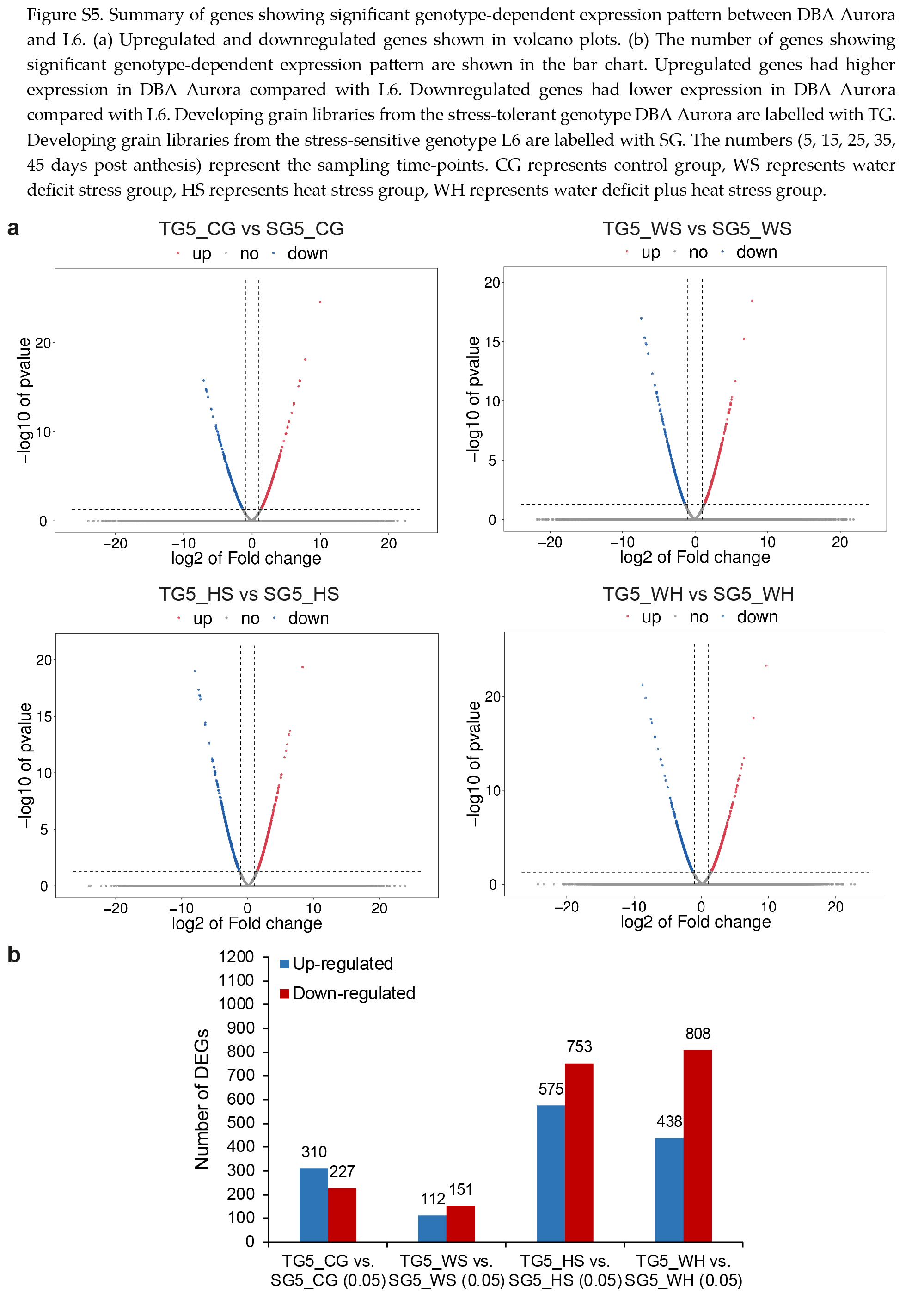

Supplement: Supplementary file 1 [file ijms-21-07772-s001.zip › Supplementary files Proof Part 1 out of 3/Figure S5.jpg]

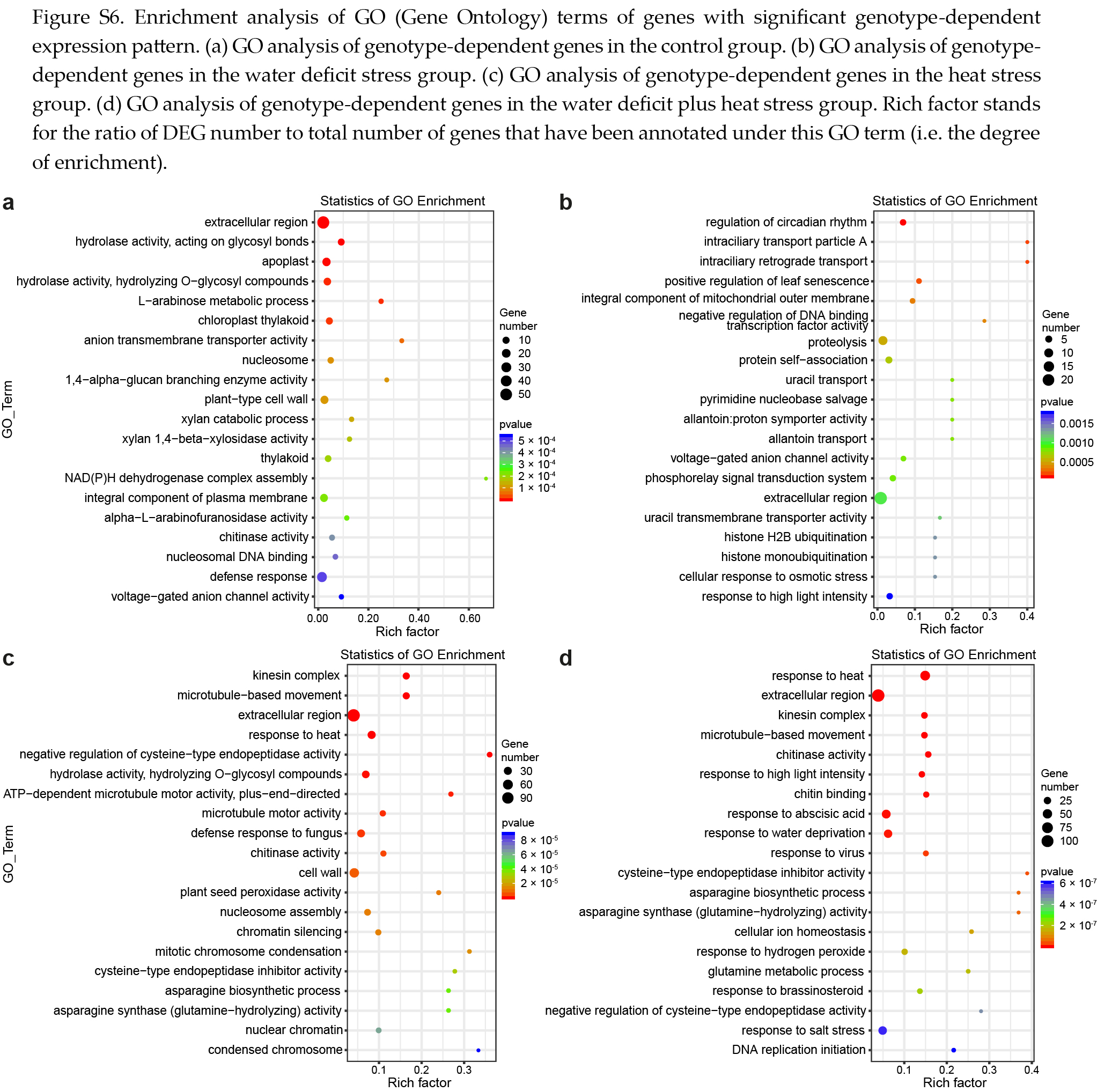

Supplement: Supplementary file 1 [file ijms-21-07772-s001.zip › Supplementary files Proof Part 1 out of 3/Figure S6.jpg]

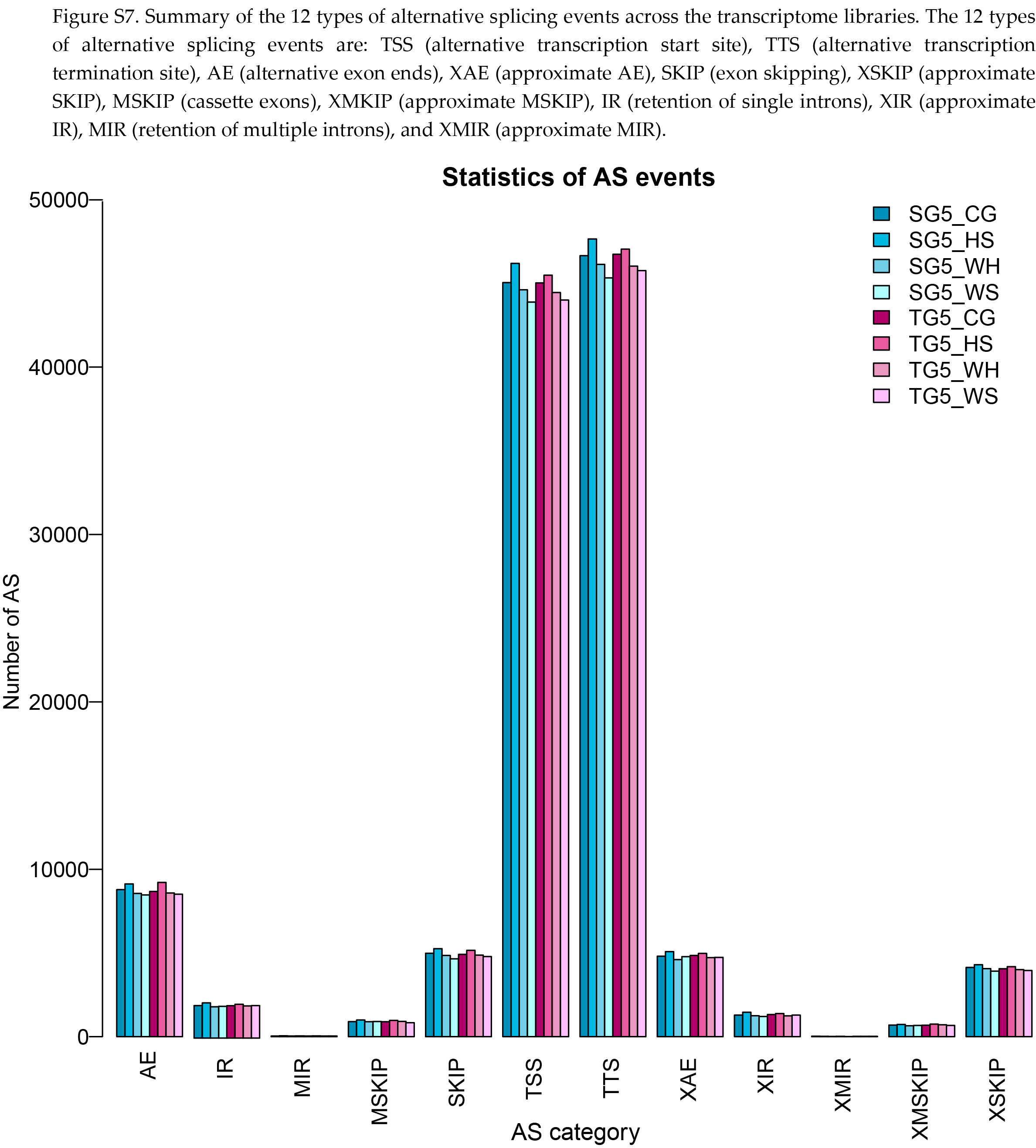

Supplement: Supplementary file 1 [file ijms-21-07772-s001.zip › Supplementary files Proof Part 1 out of 3/Figure S7.jpg]

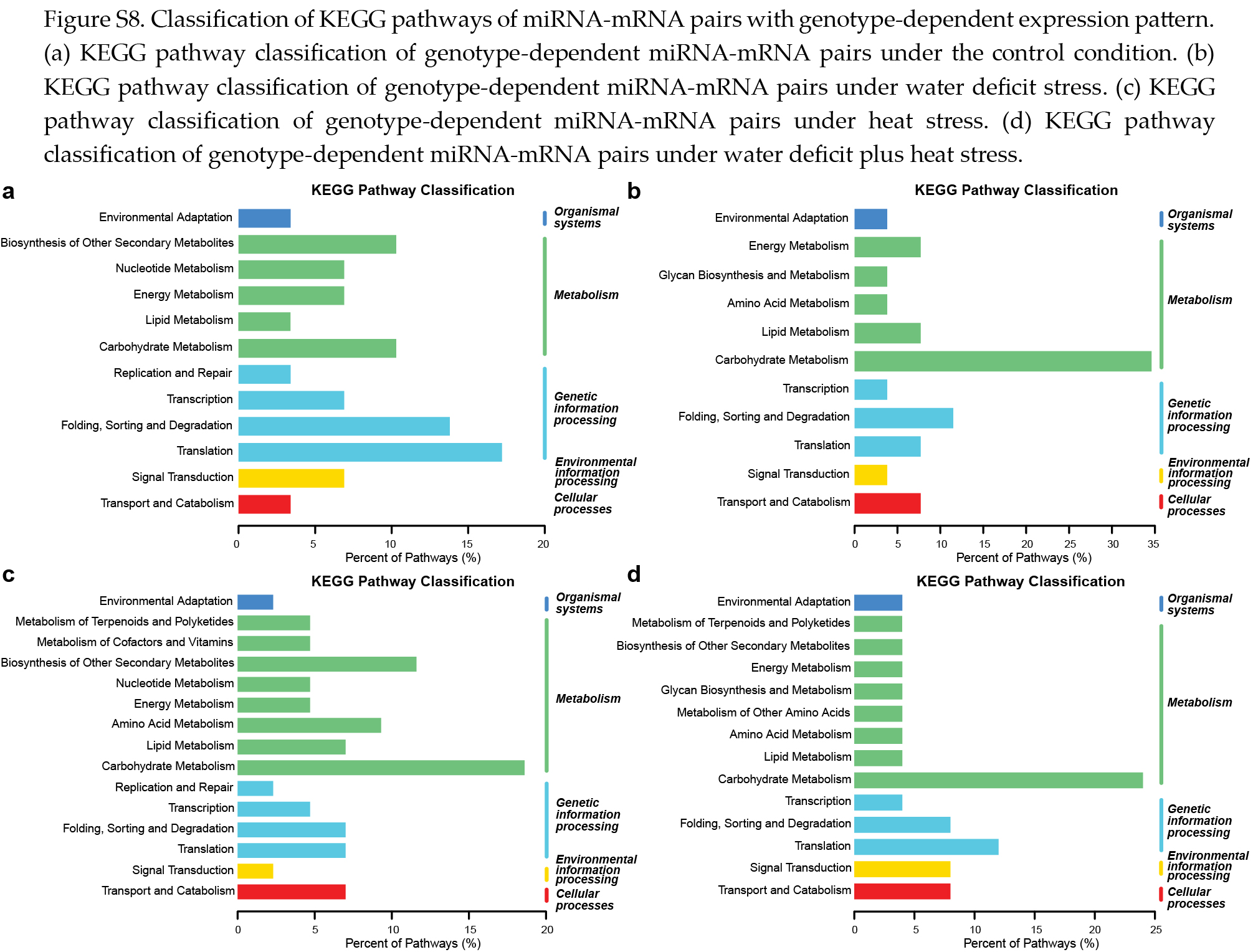

Supplement: Supplementary file 1 [file ijms-21-07772-s001.zip › Supplementary files Proof Part 1 out of 3/Figure S8.jpg]

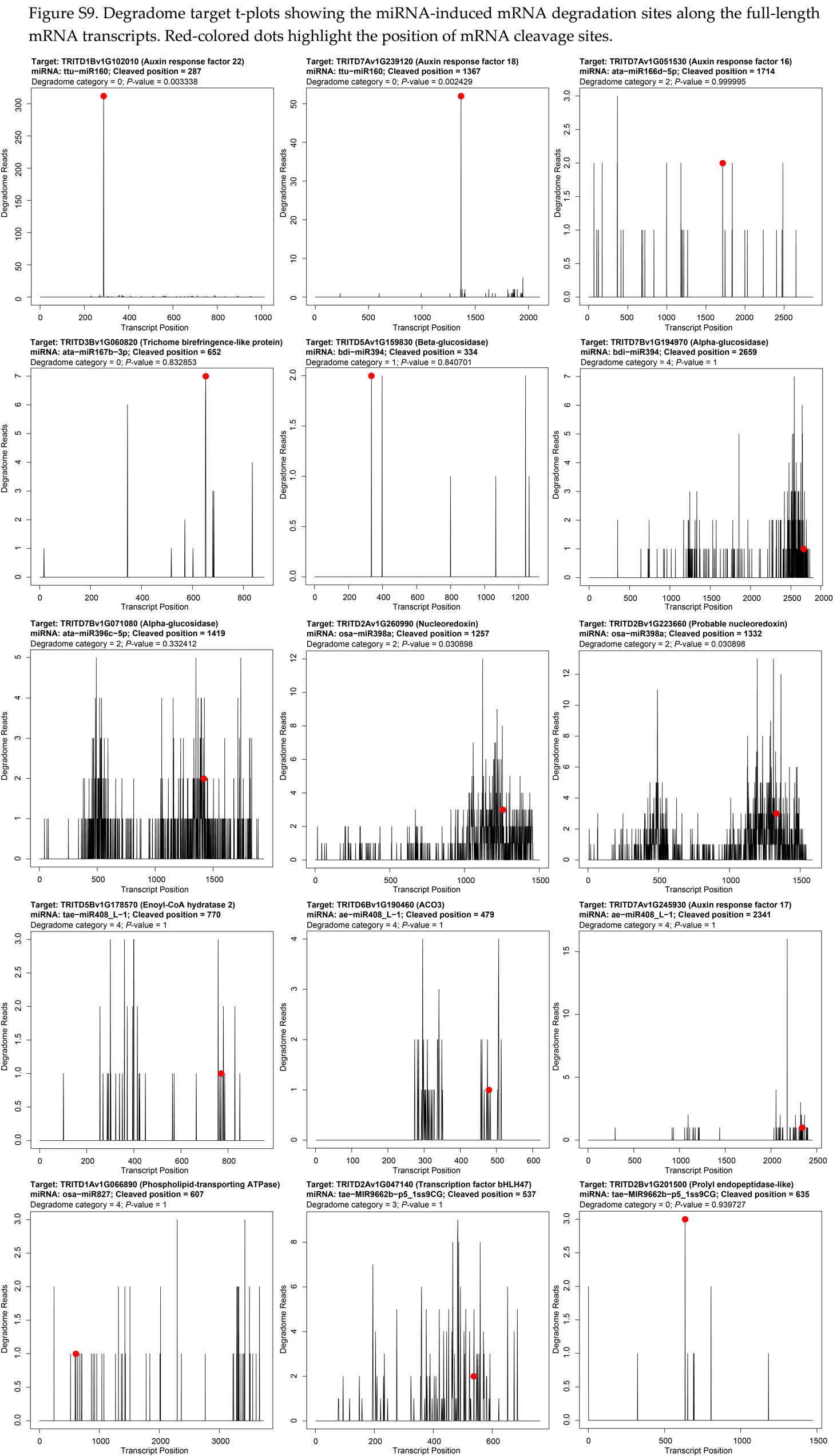

Supplement: Supplementary file 1 [file ijms-21-07772-s001.zip › Supplementary files Proof Part 1 out of 3/Figure S9.jpg]
